# Innovative γ‐Oryzanol and KC2 Based Lipid Nanoparticles: OryKL Platform Provides Safe and Efficient In Vivo mRNA Delivery

**DOI:** 10.1002/smll.202511946

**Published:** 2026-03-18

**Authors:** Pengkai Shi, Haikun Liu, Ahmed Refaat, Hung Nguyen, Anne Nguyen, Kaiting Miao, Yuyang Song, Sylvain Trépout, Rico F. Tabor, Liliana de Campo, Karlheinz Peter, Mark Louis P. Vidallon, Xiaowei Wang

**Affiliations:** ^1^ Centre For Cardiometabolic mRNA Therapy Baker Heart and Diabetes Institute Melbourne Victoria Australia; ^2^ Molecular Imaging and NanoTherapeutics Laboratory Baker Heart and Diabetes Institute Melbourne Victoria Australia; ^3^ Baker Department of Cardiometabolic Health University of Melbourne Parkville Victoria Australia; ^4^ Atherothrombosis and Vascular Biology Laboratory Baker Heart and Diabetes Institute Melbourne Victoria Australia; ^5^ School of Translational Medicine Monash University Melbourne Victoria Australia; ^6^ Ramaciotti Centre for Cryo‐Electron Microscopy Monash University Clayton Victoria Australia; ^7^ School of Chemistry Monash University Clayton Victoria Australia; ^8^ BioPRIA Department of Chemical Engineering Monash University Clayton Victoria Australia; ^9^ Australian Nuclear Science and Technology Organisation (ANSTO) Lucas Heights New South Wales Australia; ^10^ Baker Department of Cardiovascular Research Translation and Implementation La Trobe University Bundoora Victoria Australia

**Keywords:** γ‐Oryzanol, Lipid nanoparticle, mRNA therapeutics, organ distribution, storage stability

## Abstract

mRNA nanotherapeutics hold immense potential for treating a wide range of diseases, but their widespread clinical adoption is limited by current lipid nanoparticle (LNP) delivery platforms, which frequently face challenges such as limited biocompatibility, immunogenic response, insufficient mRNA delivery efficacy and stringent cold‐chain requirements. In this study, we systematically screened a 20‐member lipid mixture library by substituting ionizable lipids and sterol components to identify formulations with improved physicochemical and biological profiles. A lead candidate combining γ‐oryzanol and DLin‐KC2‐DMA as LNPs, termed OryKL (or KO 12 LNPs), was identified, exhibiting spherical bleb‐type and core–shell nanostructures (∼150 nm), high mRNA encapsulation, and significantly enhanced in vitro transfection efficiency compared to cholesterol‐based controls. Intravenous administration of OryKL delivered Cre recombinase mRNA effectively across multiple organs in Ai9 reporter mice, resulting in distinct cell‐level tropism, and no detectable toxicity or inflammation, as confirmed via qPCR, organ histology, hematological assessment and liver function tests. Additionally, OryKL retained transfection potency for at least 60 days in lyophilized form with 20% (w/v) sucrose, supporting ambient‐stable storage. These findings establish γ‐oryzanol as a promising sterol alternative and position OryKL as a biocompatible, effective, and storage‐stable platform for next‐generation mRNA therapeutics.

## Graphical abstract

Identified through multi‐stage selection process, a formulation combining γ‐oryzanol and DLin‐KC2‐DMA, termed as OryKL, demonstrates unique nano‐structural features, highly efficient mRNA transfection and functional integrity during long‐term storage, enables broad biodistribution and distinct cell tropism following intravenous administration with no detectable toxicity or inflammation in murine models. This study can guide the design of applicable vectors across various disease contexts.

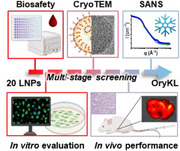

## 1 Introduction

Messenger RNA has emerged as a promising therapeutic approach for a range of diverse and therapeutically challenging diseases, offering advantages such as no risk of genome integration, high yet transient protein expression, and rapid, flexible, and scalable production [1]. However, mRNA is intrinsically unstable under physiological conditions and is prone to rapid degradation by extracellular nucleases; hence, efficient and safe delivery systems are essential to ensure mRNA protection in circulation and its subsequent uptake and translation within target cells [2]. Lipid nanoparticles (LNPs) have emerged as the most clinically advanced platform for nucleic acid delivery, with nearly 80 LNP‐based gene therapy candidates currently undergoing clinical evaluation [3]. However, several challenges continue to limit the broader application of mRNA‐LNPs in therapeutic settings beyond vaccines. These include immunogenicity, toxicity, storage constraints, and mRNA delivery inefficiencies [4]. These barriers reduce therapeutic efficacy and raise safety concerns, particularly in disease contexts where inflammation is already a key pathological feature. To address these limitations, multiple strategies have been explored, including incorporation of auxiliary components, mRNA sequence modification, and manufacturing process improvements [5]. Among these, refining the chemical composition of the LNP itself has emerged as a crucial lever for improving performance [6]. A deeper understanding of how individual LNP components influence delivery efficiency, biodistribution, and immune response is therefore essential to guide the rational design of next‐generation delivery systems.

Current LNPs are composed of four main components: (1) ionizable lipids mediate mRNA encapsulation, endosomal escape, and cellular uptake [7]; (2) phospholipids (often referred as helper lipids) stabilize the LNP structure and facilitate endosomal escape [7]; (3) cholesterol improves membrane rigidity and contributes to particle integrity [8]; and (4) polyethylene glycol (PEG)–lipids maintain colloidal stability and extend circulation time [9]. Ionizable lipids are designed to remain neutral at physiological pH to minimize systemic toxicity and immune recognition, while becoming positively charged in the acidic endosomal environment to facilitate mRNA release into the cytosol for translation [10]. Clinically validated examples include DLin‐MC3‐DMA used in Onpattro, and ALC‐0315 and SM‐102 utilized in COVID‐19 mRNA vaccines by Pfizer and Moderna, respectively [11]. While ionizable lipids can confer adjuvant‐like properties beneficial for vaccination [9], accumulating evidence indicates that they may also activate innate immune pathways and induce pro‐inflammatory cytokine responses [12, 13], posing a safety concern for therapeutic applications that require immune quiescence, such as chronic inflammatory and cardiovascular diseases [14]. Therefore, careful selection and screening of ionizable lipids is vital to balance delivery efficacy with biocompatibility.

Cholesterol is widely used as a neutral helper lipid due to its critical role in membrane stabilization [8]. However, increasing evidence indicates that cholesterol‐containing LNPs and liposomes can influence immune cell behavior and promote pro‐inflammatory signaling, particularly in macrophage‐rich environments [15, 16, 17]. These findings have prompted interest in exploring alternative sterols that could retain or improve nanoparticle performance while mitigating adverse immunological effects. Plant‐derived sterols such as β‐sitosterol and β‐stigmasterol have shown promise in enhancing biocompatibility due to their inherent anti‐inflammatory properties, improved cellular uptake, and more efficient gene delivery in nanocarrier systems [18], offering potential advantages in disease settings where inflammation must be tightly controlled.

Another major translational hurdle is the reliance on ultra‐low temperatures, driven primarily by the inherent instability of mRNA and LNP formulations [19], increasing logistical complexity and cost and limiting scalable global distribution. Strategies, such as formulation optimization, incorporating cryoprotectants [20] and freeze‐drying (lyophilization) [21], have been explored to improve storage stability and enable handling at refrigerated or ambient temperatures. However, most lyophilization studies to date have been performed on cholesterol‐containing LNPs, with limited insight into how novel sterol substitutions would influence lyophilization outcomes.

In this context, γ‐oryzanol (ORY), a phytosterol mixture derived from rice bran oil, offers a compelling yet underutilized alternative to cholesterol in LNPs. ORY has been associated with antioxidant and anti‐inflammatory activity [22] and has demonstrated excellent biocompatibility in various delivery platforms. Moreover, ORY has been successfully incorporated into solid lipid nanoparticles, yielding stable colloidal structures without compromising nanoparticle integrity [23]. These characteristics suggest that ORY could suitable as a sterol component in nanocarriers and provide a rationale for evaluating its behavior in mRNA LNP systems. Importantly, ORY differs structurally from previously investigated phytosterols [18] that typically differ from cholesterol through relatively modest changes in ring saturation or side‐chain substitutions. ORY consists of sterols esterified with bulky ferulate moieties, introducing substantial changes in molecular volume and polarity that are expected to fundamentally alter lipid packing within LNPs.

In this study, we report the systematical evaluation of 20 formulations comprising a fixed helper lipid and PEG‐lipid, with varying combinations of ionizable lipids and sterols. Our comprehensive, multi‐stage screening process included structural characterization, in vitro transfection performance, and biosafety assessments to identify lead candidates. Among these, the formulation combining ORY and DLin‐KC2‐DMA as LNPs, termed OryKL (or KO 12 LNPs), exhibited superior biocompatibility and transfection efficiency compared to conventional cholesterol‐based systems. We further validated OryKL in vivo, demonstrating efficient mRNA delivery, broad biodistribution, and a favorable safety profile in murine models. Importantly, OryKL retained high transfection efficacy after lyophilization and storage for up to two months when formulated with 20% (w/v) sucrose, underscoring their potential to overcome cold chain limitations. Altogether, our findings establish ORY as a promising alternative sterol for next‐generation LNPs and position OryKL as a robust and translationally relevant platform for mRNA therapeutics. This study provides critical design principles for advancing the field toward safer, more efficient, and storage‐stable nanocarriers.

## 2 Results and Discussion

### 2.1 Pre‐Formulation Lipid Screening Reveals Cytocompatibility and Hemocompatibility of γ‐Oryzanol Formulations

Currently, all approved mRNA LNP products contain 1,2‐distearoyl‐*sn‐*glycero‐3‐phosphocholine (DSPC) as the helper lipid and cholesterol as the primary sterol, albeit with differing ionizable and PEG‐lipid components [24]. To tailor LNPs for therapeutic applications beyond vaccines, particularly for systemic intravenous administration for chronic disease treatment, a careful redesign of LNP composition is necessary to balance mRNA delivery efficiency, circulation time, and biocompatibility [25]. In this study, we focused on evaluating the impact of ionizable lipid and sterol substitutions while keeping other components constant to enable direct comparison across formulations. We selected three clinically approved ionizable lipids, ALC‐0315 (ALC), SM‐102 (SM), and DLin‐MC3‐DMA (MC3), alongside two well‐characterized experimental lipids, C12‐200 (C12) and DLin‐KC2‐DMA (KC2). Four sterols were tested: cholesterol (CHOL), the conventional sterol as control, β‐stigmasterol (STIG), β‐sitosterol (SITO), and γ‐oryzanol (ORY). The helper lipid DSPC and the PEG‐lipid 1,2‐distearoyl‐*sn*‐glycero‐3‐phosphoethanolamine‐*N*‐[methoxy(polyethylene glycol)‐2000] (DSPE‐PEG2000) were used in all formulations at a fixed molar ratio (52.0:38.5:8.0:1.5 ionizable lipid: sterol: DSPC: DSPE‐PEG2000). DSPE‐PEG2000 was chosen over the shorter‐chains 1,2‐dimyristoyl‐*sn*‐glycero‐3‐methoxypolyethylene glycol‐2000 (DMG‐PEG2000) due to its improved anchoring stability and slower PEG desorption from the LNP surface, a property that is more suitable for therapeutic applications requiring extended systemic exposure, in contrast to the transient delivery kinetics preferred in vaccine settings. Pre‐formulation lipid mixtures were prepared using simple thin film–hydration, followed by sonication, a robust and scalable method that facilitates size reduction and uniform lipid dispersion while enabling rapid screening of multiple formulations. Dynamic light scattering (DLS) analysis confirmed that all pre‐formulation lipid mixtures had hydrodynamic diameters in the 100–300 nm range, polydispersity between 0.1 and 0.4, and near‐neutral zeta potentials (Figure S1).

**FIGURE 1:**
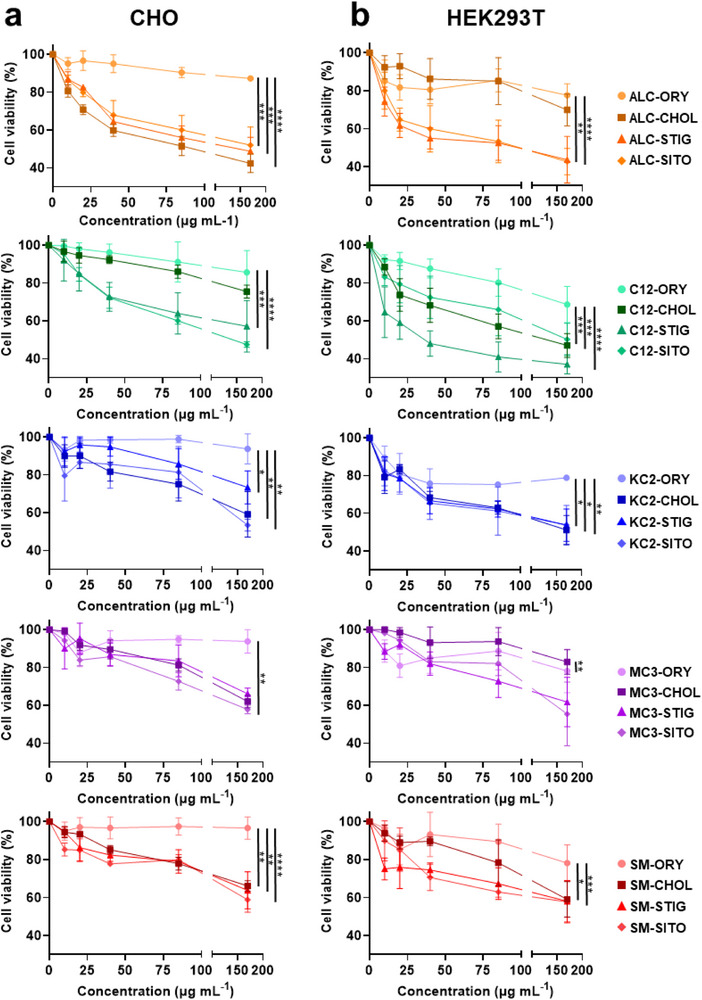
Pre‐formulation lipid screening via cytocompatibility. MTT cell viability assays using (a) CHO and (b) HEK293T cells following 24 h exposure to 20 different pre‐formulation lipid mixtures across different concentration ranges (*n* = 3 independent experiments). Values are expressed as a percentage relative to the PBS control (0 µg mL^−1^, 100% viability). Two‐way ANOVA with Geisser‐Greenhouse correction was used to evaluate the main effects of sterol type and concentration, as well as their interaction. Post hoc analysis was performed using Tukey's multiple comparisons test. Statistical comparisons reflect differences between ORY formulations with other sterol types across concentrations: *p < 0.05, **p < 0.01, ***p < 0.001, ****p < 0.0001. Complete MTT assay results with statistical comparisons are available in Figures S2 and S3.

Our initial goal was to investigate if the different pre‐formulation lipid mixtures affect biocompatibility. We first assessed cell viability across a range of concentrations (10, 20, 45, 85, and 170 µg mL^−1^) in Chinese hamster ovary (CHO) cells and human embryonic kidney (HEK293T) cells (Figure 1a,b). The selected concentration range reflects both intended and elevated doses relevant to in vitro transfection and in vivo studies and is comparable to ranges used in prior cytocompatibility studies [26, 27]. The highest concentration tested exceeds typical LNP doses reported for siRNA‐ or mRNA‐mediated gene modulation in mammalian cells by approximately 6–30‐fold [28, 29, 30]. MTT assays in CHO cells (Figure 1a; Figure S2) revealed a concentration‐dependent decline in viability for most pre‐formulation lipid mixtures. Strikingly, within each ionizable lipid series, pre‐formulation lipid mixtures incorporating γ‐oryzanol (ORY) consistently exhibited minimal cytotoxicity across all tested concentrations. In contrast, sterol variants containing CHOL, STIG and SITO showed significantly reduced cell viability, even at lower doses. Formulations containing KC2, SM, and MC3 as their ionizable lipids generally demonstrated enhanced biocompatibility, exhibiting better tolerance in treated cells. These trends were similarly observed in HEK293T cells (Figure 1b; Figure S3), confirming the robustness of the findings across cell types.

This consistently superior cytocompatibility observed in ORY‐based lipid mixtures may be attributed to the intrinsic antioxidant properties of ORY, which has been shown to reduce intracellular reactive oxygen species (ROS) and inhibit ROS‐mediated apoptotic pathways, thereby supporting cell survival and proliferation [22]. Meanwhile, CHOL‐based lipid mixtures demonstrated notable cytotoxicity at higher concentrations, likely due to their known potential to disrupt membrane integrity and interfere with cellular metabolic functions [31]. Similarly, SITO has been reported to accumulate in cell membranes, disrupt extracellular signaling and induce cell death [32]. STIG, while structurally similar, has been shown to activate both apoptosis and autophagy by inhibiting the Akt/mTOR pathway. Chronic accumulation of STIG has also been linked to cardiovascular pathologies including left ventricular dysfunction, myocardial fibrosis, macrophage infiltration, and increased mortality [33], characteristics that raise concerns about its suitability for therapeutic applications.

The observed differences in cytocompatibility between sterol variants are consistent with known sterol‐dependent biological effects reported for lipid‐based nanocarrier systems. Prior work in cholesterol‐rich liposomal systems provides important mechanistic context for interpreting the biological effects of sterol composition observed here. For example, several studies have demonstrated that cholesterol‐containing liposomes modulate immune environments [16, 34], increased secretion of pro‐inflammatory cytokines [15], enhanced macrophage activation and induce foam cell–like phenotypes in macrophages [17]. These studies highlight the potential for sterol chemistry to influence biological responses beyond nanoparticle delivery efficiency.

Although ionizable lipid‐based LNPs do not form classical phospholipid bilayers, both liposomes and LNPs typically incorporate comparable cholesterol molar fractions (approximately 30–50 mol%) [35, 36, 37, 38] and are processed through similar endo‐lysosomal pathways following cellular uptake [17, 39, 40]. Upon nanoparticle disassembly, cholesterol enters endogenous cellular lipid‐handling pathways, where it may accumulate or be metabolized into oxysterols with recognized immunomodulatory activity, particularly in macrophages [17]. While direct evidence for cholesterol‐driven immunomodulation in LNPs remains more limited than in liposomal systems, these shared features support the use of liposome studies as mechanistic references for conserved aspects of cholesterol handling rather than as structural analogues [41]. These considerations are especially relevant in cardiovascular disease contexts, where macrophage lipid loading, oxidative stress, and inflammatory signaling play central roles in disease progression [42], providing motivation for exploring alternative sterols such as γ‐oryzanol in LNP formulations.

Given that the lipid mixtures were designed for systemic delivery inevitably interact with blood components, we next assessed hemocompatibility across the same concentration range used in cytotoxicity assays. The pre‐formulation lipid mixtures were incubated with freshly collected human whole blood, and hemolytic potential was evaluated by (1) measuring hemoglobin release spectrophotometrically as an indicator of red blood cell (RBC) hemolysis, and (2) performing blood counts using a Sysmex automated hematology analyzer (Figures S4–S8). Hemolysis levels below 5% are generally considered acceptable for nanocarriers in systemic applications [43]. Across all particles and concentrations tested (Figures S4–S8), no substantial hemoglobin release was detected, and RBC, white blood cell (WBC), and platelet counts remained unchanged. These findings indicate that the tested pre‐formulation lipid mixtures are hemo‐compatible and support their favorable safety profile for systemic therapeutic use.

### 2.2 γ‐Oryzanol Formulations Show Enhanced Transfection Efficiencies

Based on the cytocompatibility and hemocompatibility profiles, pre‐formulation lipid mixtures that maintained ≥ 75% viability across the tested concentrations were prioritized for further development. Among the five sterol‐ionizable lipid combinations assessed, KC‐ORY and SM‐ORY were advanced because they displayed significantly improved tolerability relative to cholesterol and other phytosterols, while also exhibiting behavior distinct from the conventional cholesterol system and from each other. This aligned with the central objective of the study, which was to explore sterol chemistries that introduce new biological and structural profiles rather than simply replicate established formulations. STIG‐ and SITO‐containing variants did not meet the ≥ 75% viability requirement and were therefore not advanced, consistent with reports describing less favorable biological responses associated with these sterols [32, 33]. To our knowledge, ORY‐containing LNPs have not previously been reported for mRNA delivery. Cholesterol formulations, KC‐CHOL and SM‐CHOL, were therefore retained as reference benchmarks given their established role in clinically used nucleic acid nanomedicines [8].

In the following stage, LNP formulations derived from the selected pre‐formulation lipid mixtures were prepared using microfluidic mixing with a NanoAssemblr Ignite, enabling precise and rapid LNP assembly. The resulting “proto‐particle” dispersions were dialyzed against PBS, yielding the final LNPs for downstream testing. Using a representative formulation KC2 CHOL, we confirmed that increasing the total flow rate (8, 10, and 12 mL min^−1^) with a fixed flow ratio (3:1 aqueous‐to‐ethanolic) results in a reduction in particle size (Figure S9), providing a foundation for controlled size tuning. To systematically incorporate size as a variable, flow rates of 8 and 12 mL min^−1^ were selected to produce two distinct particle sizes per formulation. For clarity, formulations are hereafter designated by the identity of the ionizable lipid, the sterol, and the fabrication flow rate (Table 1). SM with ORY (SO) LNPs were excluded from this stage due to poor performance in the initial transfection trials.

**TABLE 1:** Nomenclature of mRNA LNP formulations tested for transfection efficiency.

Formulation	Ionizable lipid^*^	Sterol	Total flow rate (mL min^−1^)
KC 8	KC2	CHOL	8
KC 12	KC2	CHOL	12
SC 8	SM	CHOL	8
SC 12	SM	CHOL	12
KO 8	KC2	ORY	8
KO 12	KC2	ORY	12

^*^ KC2 = Dlin‐KC2‐DMA; SM = SM102.

**FIGURE 2:**
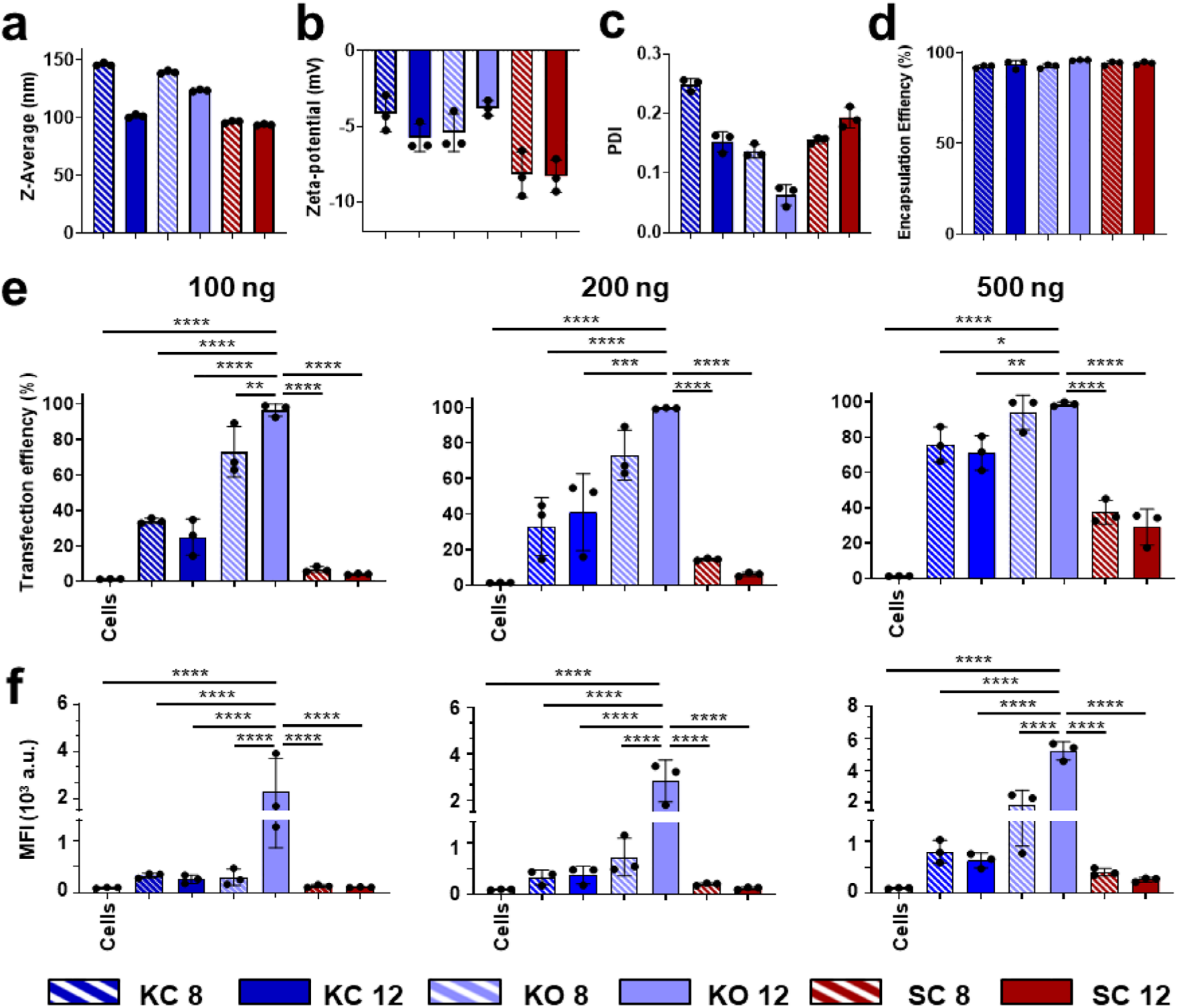
Physicochemical properties and transfection efficiencies of mRNA LNPs. Physicochemical properties of different LNP formulations, including (a) hydrodynamic diameters (Z‐average), (b) zeta potential, (c) PDI, and (d) encapsulation efficiency. CHO cells were treated with mRNA LNPs for 24 h at doses of 100, 200, and 500 ng eGFP mRNA per 10^5^ cells. Flow cytometry analysis showing the (e) CHO cell population expressing eGFP, indicating transfection efficiency, and (f) MFI of eGFP in transfected cells, indicating protein expression levels. Bar graphs are shown as mean ± SD from independent experiment (*n* = 3). Statistical significance was tested using one‐way ANOVA, followed by Dunnett's multiple comparisons test (comparing all treatment groups). Statistical comparisons reflect differences between KO 12 formulation with other groups (e and f), *p < 0.05, **p < 0.01, ***p < 0.001, ****p < 0.0001.

DLS analysis in Figure 2 confirmed that all fabricated LNPs possessed hydrodynamic diameters below 150 nm with narrow size distributions, as indicated by polydispersity indexes (PDI) values below 0.3. Zeta potential measurements indicated a near neutral surface charge across all formulations, consistent with effective removal of excess cationic charge via the post‐formulation dialysis step. All LNPs exhibited high eGFP mRNA encapsulation efficiencies (EE > 90%), indicating the robustness of the selected lipid compositions and fabrication parameters in capturing and retaining the mRNA payload. Cytocompatibility profile of lead KO 12 and its cholesterol‐based KC 12 LNP formulations also corroborate with pre‐formulation lipid mixtures (Figure S10).

To evaluate the mRNA transfection efficiency, CHO cells were treated with varying concentrations of LNPs, and transfection outcomes were quantified via flow cytometry. The percentage of eGFP‐positive cells in Figure 2e reflects transfection efficiency, while the mean fluorescence intensity (MFI) in Figure 2f provides a measure of protein expression level in the transfected cells. All tested formulations showed dose‐dependent increases in both parameters, with KO 12 LNPs showing the highest performance across all concentrations, and KO 8 LNPs exhibited moderately lower performance, though still surpassed SM‐based and CHOL‐based counterparts. For KO 12, this trend was further supported by transfection assays conducted at lower mRNA doses (Figure S11).

**FIGURE 3:**
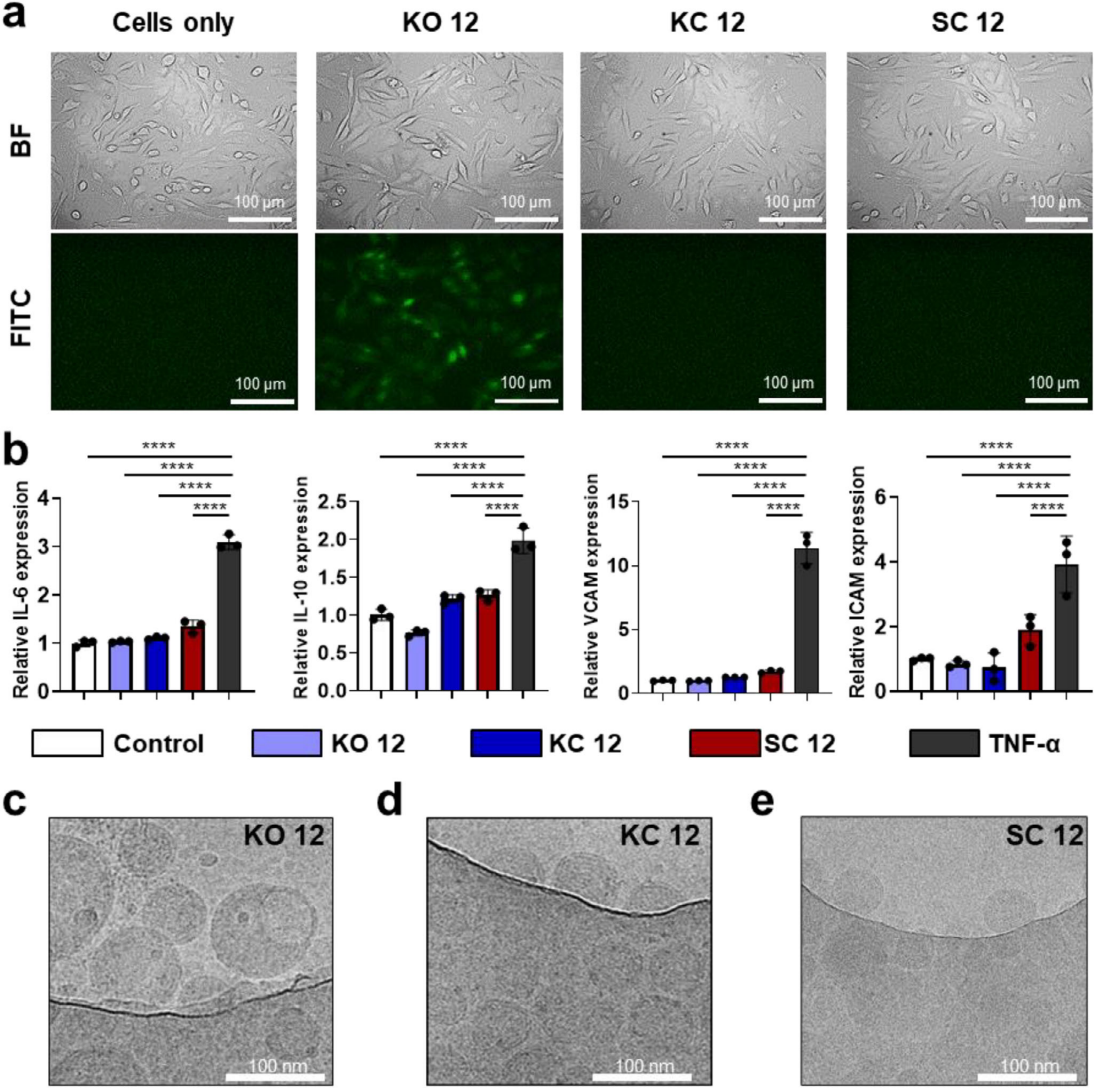
Evaluation of mRNA translation, in vitro immunogenicity, and nanostructure of mRNA LNP formulations. (a) Representative optical photomicrographs of LNP‐transfected CHO cells with the brightfield (BF) images showing general cell morphology and FITC images showing eGFP fluorescence (green). (b) Relative quantification of expression levels of pro‐inflammatory IL‐6, anti‐inflammatory IL‐10, adhesion molecules VCAM‐1 and ICAM‐1 following 24 h induction of SVEC4‐10 cells with different LNP formulations at an eGFP mRNA dose of 200 ng per 10^5^ cells and with TNF‐α (10 ng per 10^5^ cells) as the positive control. Bar graphs are shown as mean ± SD from independent experiment (*n* = 3) using one‐way ANOVA (comparing means for all treatment groups in (b). Statistical comparisons reflect differences between all formulations (and control) and the TNF‐α treatment; *p < 0.05, **p < 0.01, ***p < 0.001, ****p < 0.0001. Representative cryoTEM images depicting the overall morphologies and internal structures of LNPs prepared at flow rate = 12 mL min^−1^: (c) KO 12; (d) KC 12; and (e) SC 12 LNPs.

These trends were further supported by epifluorescence microscopy of CHO cells (Figure 3a). KO 12 LNPs elicited sufficient eGFP expression to be visualized by microscopy, consistent with the MFI values (∼5000). Other LNP‐treated groups (KC 12 and SC 12 LNPs) did not yield detectable fluorescence, despite comparable cell morphology under brightfield imaging. This absence of morphological changes confirms that all formulations were well tolerated, in agreement with the cytocompatibility data from Figure 1. While functional reporter expression confirms productive mRNA delivery, the intracellular distribution of mRNA following endosomal escape was not directly examined. The superior transfection results of KO 12 LNPs may arise from multiple contributing factors (i.e., the unique chemical structures of ORY being recognized differently by cells) and/or colloidal structure effect (i.e., overall particle size, nano‐ and ultrastructures, underlying lipid phase transformations), which potentially facilitate more efficient cellular uptake, mRNA delivery and/or endosomal escape. These factors and others, which might also influence their transfection potency, will require further investigation through detailed biophysical and mechanistic studies.

### 2.3 In Vitro qPCR Readouts Indicate Low Immunogenicity

mRNA‐loaded LNPs are known to induce cytokine expression in transfected cells, a property that, can enhance vaccine efficacy but may pose significant risks in therapeutic settings, particularly in chronic inflammatory conditions and cardiovascular diseases. Hence, characterizing the immunogenic profile of LNP formulations is essential to assess their suitability for systemic therapeutic use.

To evaluate the in vitro immunogenicity of selected LNPs, SVEC4‐10 endothelial cells were transfected with KO 12, KC 12, and SC 12 formulations at an eGFP mRNA dose of 200 ng per 10^5^ cells, and cytokine gene expression was measured via quantitative PCR (qPCR) at 24 h post‐treatment as shown in Figure 3b. A cytokine panel was selected to include both pro‐ and anti‐inflammatory markers, such as interleukin‐6 (IL‐6), interleukin‐10 (IL‐10), and the adhesion molecules VCAM‐1 and ICAM‐1. Tumor necrosis factor‐alpha (TNF‐α, 10 ng per 10^5^ cells) served as a positive control for cytokine induction. All three LNP formulations showed minimal induction of cytokine or adhesion molecule transcripts. In endothelial cells such as SVEC4‐10, TNF‐α induces IL‐10 via a self‐limiting feedback mechanism using nuclear factor kappa B cells dependent pathway to regulate inflammation [44]. These results suggest a low immunogenic potential, supporting the clinical relevance of these LNPs, particularly the novel KO 12, for use in chronic and inflammation‐sensitive therapeutic applications.

### 2.4 Cryogenic Transmission Electron Microscopy Reveals the Internal Structures of KO 12 LNPs

To gain further insight into the morphology and internal structures of these LNPs, cryogenic transmission electron microscopy (cryoTEM) was performed on KC 12, KO 12, and SC 12. All formulations displayed spherical morphologies with no visible aggregation (Figure 3c–e). Each particle showed a well‐defined exterior structure (Figure S9a) with at least two visible lamella layers (lipid‐rich layers appearing as dark bands) with 3–4 nm thickness, separated by 2–3 nm interlamellar spacings or gaps (lighter regions between the bands). Internal structures varied across the formulations. KC 12 and SC 12 showed internal features resembling entangled strings with random orientations. In contrast, KO 12 LNPs exhibited a unique dual‐phase, bleb‐like internal architecture (Figure S9b), characterized by a darker, speckled spherical protrusion attached to a larger, uniformly grey semi‐spherical phase. The higher electron contrast in the smaller bleb regions suggests enrichment in mRNA, likely due to the higher electron density associated with phosphorus atoms in the nucleic acid backbone. The larger semi‐spherical region appears lipid‐rich and mRNA‐poor, suggesting phase separation within the nanoparticle.

The emergence of this dual‐phase morphology in KO 12 can be rationalized by the molecular structure of γ‐oryzanol. Although a fixed molar fraction of sterol (38.5 mol%) was used across all formulations, γ‐oryzanol is substantially bulkier than cholesterol (603 g mol^−1^ average vs. 387 g mol^−1^), with a calculated molecular volume of ∼0.91 nm^3^ compared to ∼0.61 nm^3^ for cholesterol. These differences are expected to influence sterol–lipid packing within the LNPs and likely contributes to the larger average particle size of KO 12 relative to KC 12 and SC 12. γ‐Oryzanol is a mixture of *trans*‐ferulic acid esters of sterols, predominantly cycloartenyl ferulate, 24‐methylenecycloartanyl ferulate, and campesteryl ferulate, which collectively possess rigid sterol backbones appended with bulky ferulate moieties [45]. These structural features introduce both increased hydrophobic volume and local polarity, reducing packing compatibility with ionizable lipids and phospholipids compared to cholesterol. Consequently, γ‐oryzanol is likely to promote segregation into different lipid‐rich domains, leading to enhanced phase heterogeneity and bleb‐like, water‐rich regions in a subset of the LNP population, as suggested by cryoTEM observations of KO 12 LNPs.

Notably, bleb structures were not observed in KC 12 LNPs, where we have previously shown core–shell spherical architecture with cholesterol distributed across both core and shell phases [46]. The appearance of blebs in KO 12 therefore represents a fundamental deviation from the canonical cholesterol‐stabilized LNP structure, rather than a minor morphological variation. To the best of our knowledge, γ‐oryzanol has previously been used only as a payload in non‐mRNA LNP nanocarrier systems [23, 47, 48], and this work represents its first incorporation into mRNA LNPs as a sterol component. Compared with other phytosterol substitutions that mainly affect membrane rigidity or external particle morphology, γ‐oryzanol is associated with internal phase heterogeneity in KO 12 LNPs. Prior studies [18, 49] with sterols such as sitosterol or stigmasterol have linked altered packing to non‐spherical morphologies, whereas the present system displays internal compositional compartmentalization that may influence mRNA distribution.

Previous studies have associated bleb structures with enhanced transfection potency both in vitro and in vivo, potentially due to improved retention of mRNA integrity and more efficient release kinetics from LNPs [50, 51]. The distinct dual‐phase morphology observed in KO 12 LNPs may therefore play a functional role in facilitating mRNA delivery. However, it remains unclear whether these advantages stem solely from structural characteristics or also involve biological interactions unique to γ‐oryzanol or other sterol components. Further mechanistic studies are warranted to elucidate the contributions of both physical architecture and lipid composition. Collectively, these findings highlight that both the formulation composition and fabrication parameters critically influence LNP particle size, morphology, and internal structure, all of which may ultimately modulate mRNA localization, stability, and delivery efficiency.

### 2.5 In Vivo Efficacy and Safety Profile of OryKL (KO 12 LNPs) during Systemic mRNA Delivery

Through a systematic multi‐stage evaluation, KO 12 LNPs, hereafter termed OryKL, were nominated as the lead formulation based on predefined translational criteria, including reproducible physicochemical properties, efficient mRNA encapsulation, favorable cyto‐ and hemocompatibility, and consistent transfection across a relevant concentration range. We next assessed their capacity to deliver mRNA in vivo. Mice were administered with OryKL loaded with mCherry mRNA via either intravenous (IV) or intraperitoneal (IP) injections. Since previous studies demonstrated LNP accumulation in the liver due to its highly fenestrated and vascularized endothelium [52], we focused our initial assessment on hepatic mRNA expression. Flow cytometry of liver‐isolated cells 24 h post‐injection showed substantial mCherry expression in mice treated with OryKL, (Figure S12a) and a marked rightward shift in mean fluorescence intensity (Figure S12b), compared to control animals. IV administration resulted in higher mCherry expression compared to IP, indicating superior systemic delivery via the IV route. These findings validated the capacity of OryKL LNPs to deliver functional mRNA to liver cells in vivo and guided subsequent biodistribution and safety studies via IV administration.

#### 2.5.1 In Vivo Biodistribution of Intravenously Administered NanoLuc and Cre Recombinase mRNA‐Loaded OryKL

In this study, organ‐specific mRNA delivery at a functional level, we performed in vivo biodistribution using OryKL loaded with mRNA encoding for NanoLuc reporter. NanoLuc bioluminescence provides a sensitive readout of successful cytosolic mRNA delivery and protein translation, rather than physical mRNA accumulation alone [53, 54, 55]. Consistent with other reported LNP formulations, OryKL predominantly delivers mRNA mainly to the liver (Figure S13) and further provides an independent functional confirmation of the hepatic mRNA expression observed in the flow cytometry result in the above in vivo mCherry experiments.

**FIGURE 4:**
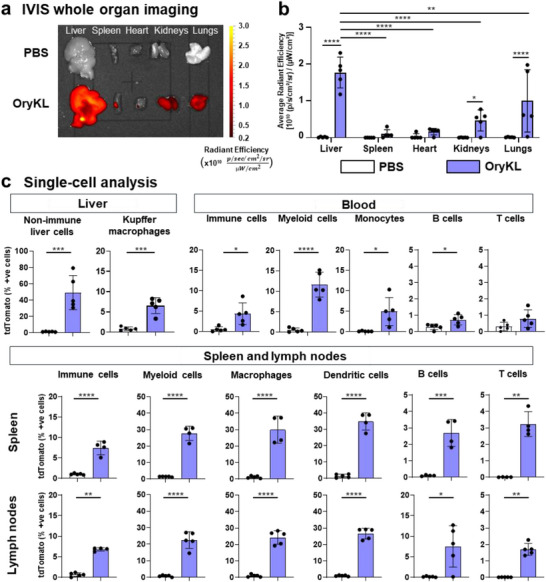
In vivo mRNA delivery and biodistribution of OryKL in Ai9 mice. (a) Representative IVIS fluorescence photomicrographs of isolated major organs of Ai9 mice 72 h post‐treatment with Cre recombinase mRNA‐loaded OryKL and with PBS (control). Color scale shows the fluorescence signal intensities as average radiant efficiency in 10^10^ [p/s/cm^2^/sr] / [µW/cm^2^]. (b) Bar graphs showing the quantitative analysis using average radiant efficiency in the fluorescence photomicrographs of isolated major organs. (c) Flow cytometry data showing the cell tropism of OryKL in collected major organs: liver, blood, spleen, and lymph nodes. Bar graphs are shown as mean ± SD from independent animals (*n* = 4–5), using two‐way ANOVA (comparing means for all organs and treatment groups in b and two‐tailed, t test in c, *p < 0.05, **p < 0.01, ***p < 0.001, ****p < 0.0001.

To extend the tracking of functional mRNA delivery in vivo at both organ and cell levels, we employed the Ai9 reporter mouse model, which expresses a robust red fluorescent tdTomato protein upon transfection with Cre recombinase mRNA. OryKL loaded with Cre mRNA were administered via tail vein injections. At 72 h post‐injection, fluorescence imaging of major organs revealed tdTomato expression in the liver, lungs and kidneys (Figure 4a,b), indicating effective delivery and translation of Cre mRNA in multiple tissues. The broader distribution in the tdTomato system may reflect reporter amplification, biological kinetics, or redistribution of transfected cells. Therefore, the current data support predominant hepatic expression but does not establish intrinsic organ‐specific tropism. The pronounced hepatic localization of OryKL is consistent with established features of liver physiology that favor nanoparticle accumulation following systemic administration. Additionally, rapid adsorption of apolipoprotein E (ApoE) onto the LNP surface enhances hepatocyte uptake via low‐density lipoprotein (LDL) receptors [52]. Flow cytometry analysis revealed that ∼50% of non‐immune liver cells (CD45^−^), such as hepatocytes, consistent with ApoE‐mediated pathways reported for ionizable lipid‐based LNPs [56], and liver sinusoidal endothelial cells (LSECs), expressed tdTomato (Figure 4c), confirming efficient delivery to key hepatic cell types. Hepatocytes constitute the main functional liver cell types, mediating critical processes including protein synthesis, detoxification and lipid metabolism. While LSECs facilitate LNP transfer between blood and hepatocytes via their fenestrated structures, hepatocytes exhibit robust LNP uptake and potent mRNA translation, largely mediated by LDL receptor‐dependent pathways, making them a predominant target for LNP‐based therapeutic application [52]. Interestingly, Kupffer cells (CD45^+^CD11b^+^CD68^+^) showed limited tdTomato expression (∼6% cells), consistent with their known role in nanoparticle sequestration and innate immune activation [57]. Previous studies have demonstrated that modifying sterol components within LNPs can reduce Kupffer cell uptake and enhance delivery to non‐immune hepatic cells [58, 59]. The low tdTomato expression observed in Kupffer cells suggests that incorporation of γ‐oryzanol may modulate cellular tropism by reducing uptake by hepatic immune cells. This feature is particularly advantageous in the context of chronic diseases, where targeted delivery to non‐immune cells and avoidance of immune activation are critical for maximizing therapeutic efficacy while minimizing the risk of exacerbating underlying inflammation.

Beyond hepatic tropism, OryKL also displayed strong uptake by immune cells in secondary lymphoid organs. Flow cytometry revealed tdTomato expression in ∼28% of myeloid cells (CD45^+^CD11b^+^) in the spleen, and ∼22% in lymph nodes, with moderate levels (∼11.5%) in circulating blood (Figure 4c). The systemic bioavailability of OryKL observed is likely attributable to the incorporation of a strongly anchored, slow‐desorbing DSPE‐PEG2000 lipopolymer in LNP formulation, which is expected to extend LNP circulation time, reduce opsonization and enhance accumulation in tissue‐resident immune cells [56]. The PEG‐lipid composition, particle size, and surface charge are known to influence lymphatic drainage and uptake [60, 61]. Further analysis showed high transfection levels (∼20%–40%) in macrophages (CD45^+^CD11b^+^CD68^+^) and dendritic cells (CD45^+^CD11b^+^CD11c^+^) in both lymph nodes and spleens. These patterns align with other reports investigating LNP biodistribution at the cellular level [62, 63, 64], and reflect the high phagocytic nature of these immune subsets. In contrast, tdTomato expression in lymphocytes (T and B cells) was negligible, indicating preferential uptake by antigen‐presenting cells rather than adaptive immune cells.

#### 2.5.2 In Vivo Safety Profile of OryKL

Since OryKL represents a novel LNP formulation, it is essential to evaluate its in vivo immunogenicity and biosafety profile to assess its suitability for clinical translation. LNPs are known to induce innate immune responses, often via toll‐like receptor (TLR) and retinoic acid‐inducible gene (RIG)‐I‐like receptors (RLRs) [65]. To this end, we quantified the expression of TLR‐ and RLR‐responsive genes in the liver, spleen, and kidney, the primary organs where OryKL successfully delivered Cre mRNA.

**FIGURE 5:**
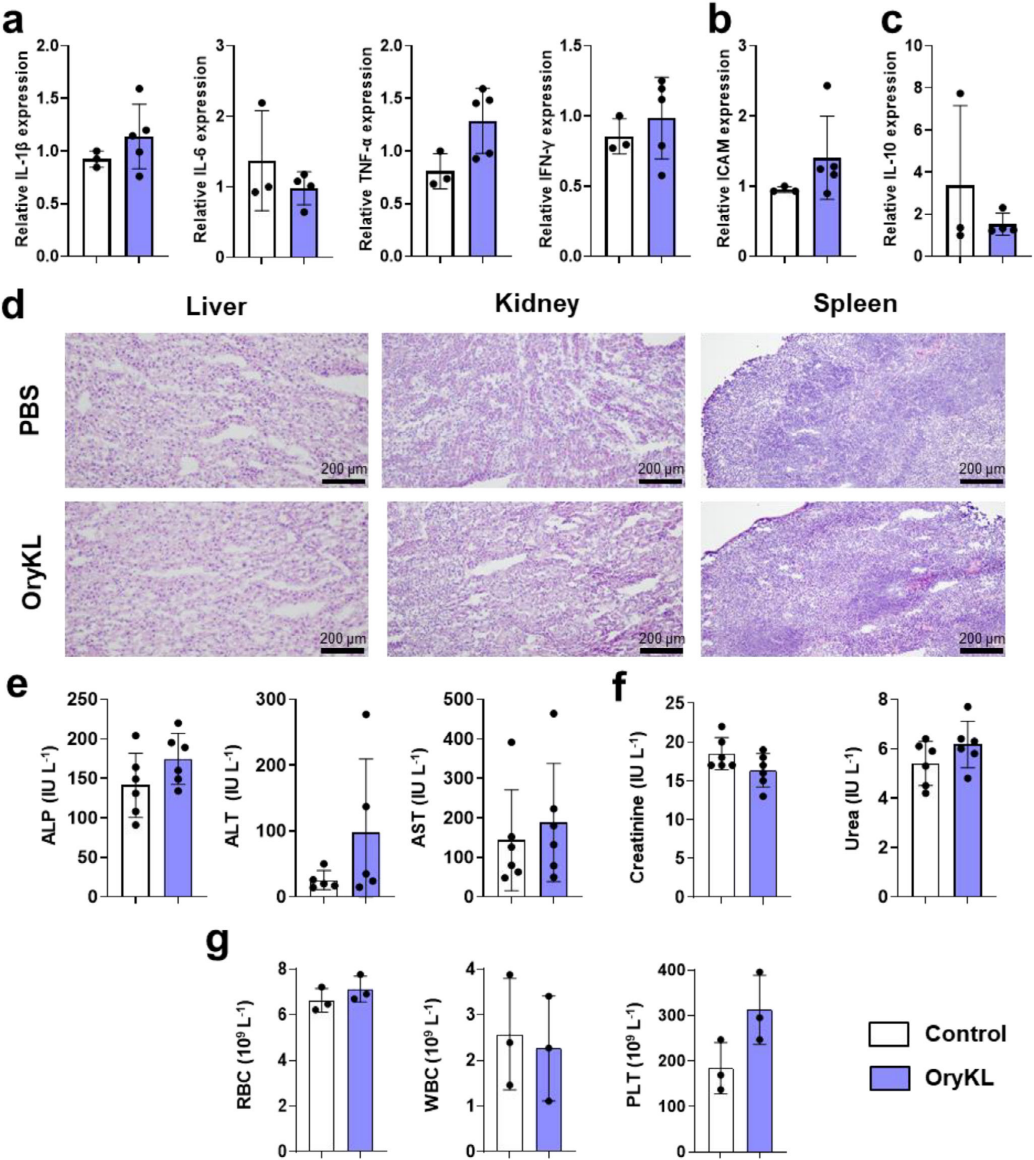
Safety profile of OryKL. (a) Relative quantification of gene expression via qPCR indicating immunogenicity of Cre recombinase mRNA‐loaded OryKL in liver cells from mice, 24 h post administration of LNPs via intravenous (IV) injection: pro‐inflammatory cytokines IL‐1 beta, IL‐6, TNF‐alpha, IFN‐gamma, *n* = 3–5; (b) adhesion molecule ICAM‐1; and (c) anti‐inflammatory cytokine IL‐10. (d) H&E staining of mouse liver, kidney and spleen from OryKL treated group and PBS control. Mouse serum analysis after OryKL injection: (e) liver function markers (ALP, ALT, and AST), *n* = 6; and (f) kidney function markers (creatinine and urea), *n* = 6; (g) Hemocompatibility test using murine blood following IV injection, *n* = 3. Bar graphs are shown as mean ± SD from independent animals (*n* ≥ 3), using two‐tailed, t test (a–c, e–g), *p < 0.05, **p < 0.01, ***p < 0.001, ****p < 0.0001.

As shown in Figure 5a–c, gene expression analysis revealed no significant upregulation of pro‐inflammatory cytokines (IL‐1β, IL‐6, TNF‐α, IFN‐γ), the adhesion molecule ICAM‐1, or the anti‐inflammatory cytokine IL‐10 in OryKL‐treated mice compared to PBS controls. Although minor increases were observed in some markers, these changes were not significant, indicating that systemic mRNA delivery by OryKL does not elicit a notable inflammatory or immunosuppressive response in our murine model.

Histological examination via hematoxylin and eosin (H&E) staining showed no evidence of tissue damage, inflammation, or pathological alterations in the liver, kidney, or spleen (Figure 5d). To further assess renal and hepatic compatibility, serum biomarkers, including alkaline phosphatase (ALP), alanine transaminase (ALT), aspartate transaminase (AST), creatinine and urea were measured (Figure 5e,f). A mild but non‐significant trend toward ALT elevation was observed, consistent with prior reports describing transient and reversible hepatocellular stress linked to certain LNP excipients, including PEG‐lipids [9]. The other major constituents, including ionizable lipids and sterol components may also influence hepatic responses, as these materials have been reported to engage innate immune pathways that are typically accompanied by cytokine and chemokine induction [14, 66]. In this study, no detectable cytokine responses or histological abnormalities were observed; the ALT trend is therefore most consistent with low‐grade, subclinical hepatic stress without overt immune activation, supporting a favorable tolerability profile under the conditions tested. While γ‐oryzanol possesses recognized anti‐inflammatory properties, our result supports the safety of the integrated lipid system rather than attributing the outcome to any single component.

Hematological parameters were also evaluated using a Sysmex automated analyzer. No significant changes were detected in red blood cells, white blood cells, or platelets (Figure 5g), supporting the hemocompatibility of OryKL following in vivo systemic administration. While comprehensive hemocompatibility and inflammatory profiling were performed, and no acute systemic immune activation was detected, PEG‐specific immune responses and complement activation were not directly assessed in this study.

Collectively, these findings demonstrate that OryKL exhibits a favorable biosafety profile in vivo. The absence of histological or biochemical abnormalities indicates low immunostimulatory potential, even in highly perfused and immunologically active organs. The observed high liver transfection efficiency, coupled with minimal Kupffer cell activation and no hepatocellular injury, supports a broad therapeutic window. This positions OryKL as a promising platform for applications requiring targeted, transient mRNA expression in the liver or immune tissues. Importantly, OryKL is not intended as a universal replacement for cholesterol in mRNA LNP formulations. Cholesterol remains highly effective, particularly in vaccines where engagement of innate immune pathways can enhance adjuvant activity. Instead, LNP design should be tailored to the biological context and therapeutic objective. The rationale for OryKL is to support expansion of LNP technology into therapeutic settings characterized by pre‐existing inflammation, where additional immune stimulation may reduce tolerability during systemic or repeated administration. Such applications include transient expression of anti‐inflammatory or anti‐thrombotic proteins, and therapeutic interventions for liver‐associated pathologies, including hepatocellular carcinoma and fatty liver disease [52]. Future work will aim to extend safety evaluation to additional immunological and blood compatibility parameters, including complement activation, anti‐PEG antibody responses, and plasma protein interactions.

### 2.6 Sucrose and Trehalose Extend the Transfection Performance of OryKL Under Different Storage Conditions

Currently approved mRNA LNP formulations require ultra‐low temperature storage with cryoprotectant for preservation of stability and bioactivity [67]. However, storage performance is highly dependent on parameters such as temperature, freeze‐thaw conditions, and the choice of cryoprotectant. Sugar‐based excipients, such as sucrose and trehalose, are widely used to stabilize LNPs during freeze‐thawing and lyophilization. For example, BNT162B2 uses PBS with 20% w/v sucrose and remains stable for up to 6 months at −70°C, whereas TT3 ‐based formulations require flash‐freezing in liquid nitrogen and maintain functionality for three months with only 5% w/v sucrose or trehalose [19, 67].

**FIGURE 6:**
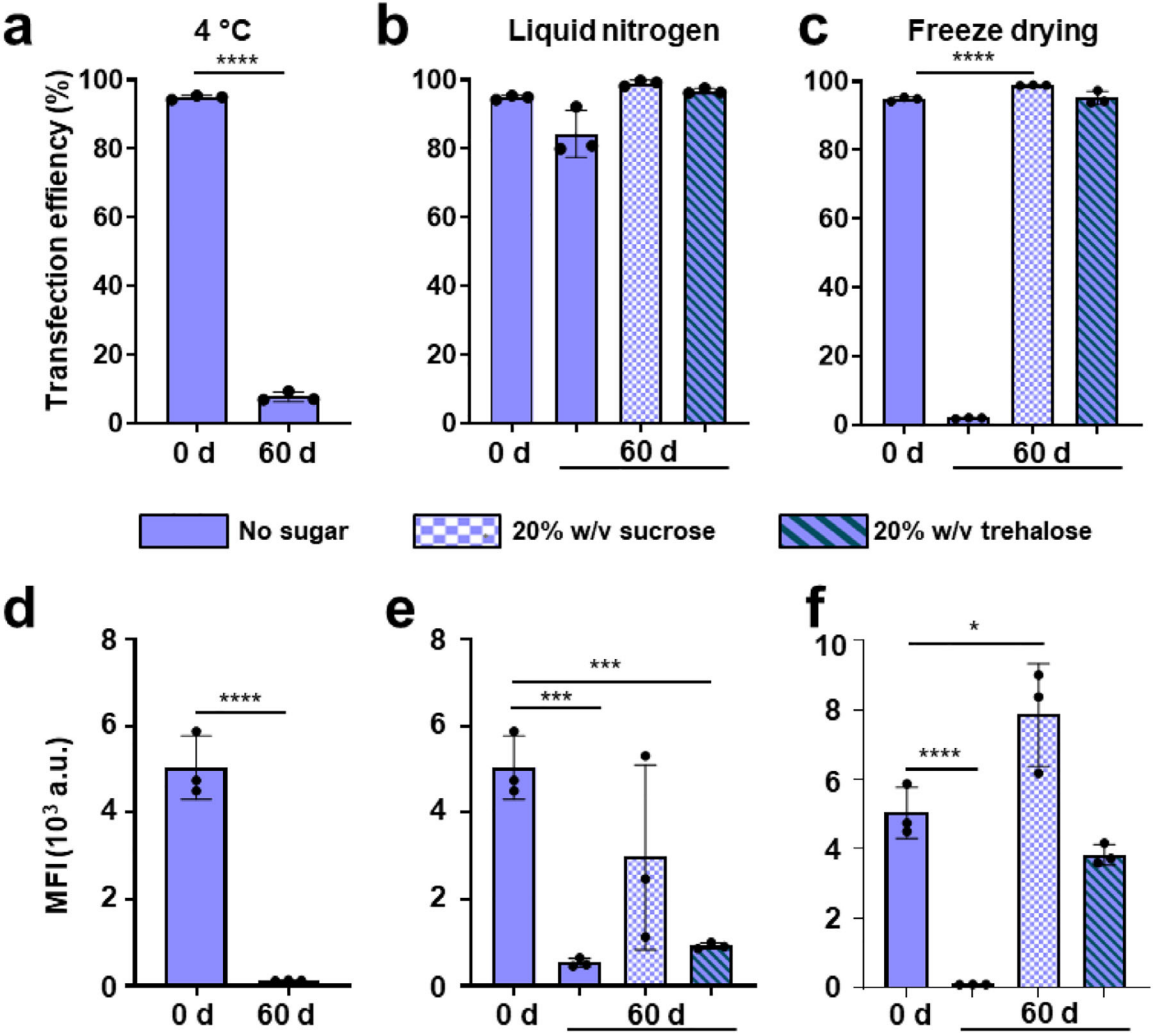
Low temperature‐storage stability of OryKL. (a–c) Transfection efficiency and (d–e) MFI data on CHO cells 24 h post‐transfection with 500 ng eGFP mRNA‐loaded OryKL per 10^5^ cells, with and without cryoprotectant, 0 d and after 60 d storage under different condition under different storage conditions: (a and d) at 4°C; (b and e) in liquid nitrogen; and (c and f) at −20°C for freeze‐dried samples. Bar graphs are shown as mean ± SD from independent experiment (*n* = 3), using one‐way ANOVA (comparing means between storage groups on 0 d and 60 d, *p < 0.05, ***p < 0.001, ****p < 0.0001.

To investigate the storage stability of OryKL, we examined the effects of different cryoprotectants (sucrose and trehalose) and storage conditions, including refrigeration at 4°C, flash freezing in liquid nitrogen, and freeze‐drying followed by storage at −20°C. Transfection efficiency and protein expression were evaluated in CHO cells at baseline (Day 0) and after 60 days of storage (Figure 6a–f). An eGFP mRNA dose of 500 ng per 10^5^ cells was used to maintain detectable transfection levels and enable assessment of potential storage‐induced decreases in LNP efficacy. At baseline, all freshly prepared OryKL samples, regardless of cryoprotectant, exhibited high transfection efficiency and MFI, consistent with earlier results (Figure S14); however, after 60 days, marked differences emerged across storage conditions. LNPs stored at 4°C (without cryoprotectants) showed a 90% decrease in transfection efficiency and undetectable eGFP expression, indicating an almost complete loss of function (Figure 6a,d). In contrast, flash‐frozen samples retained moderate functionality (Figure 6b,e). Both sucrose and trehalose conferred protection during liquid nitrogen storage, although samples without cryoprotectants showed a 10% reduction in transfection efficiency. Interestingly, only sucrose moderately preserved protein expression (MFI), while trehalose offered limited benefit, and cryoprotectant‐free samples demonstrated a marked decline in translational activity. These findings suggest that although cell uptake is somewhat preserved under cryogenic storage, cryo‐induced structural changes likely impair mRNA release or translation.

Remarkably, freeze‐drying followed by storage at −20°C (standard freezer temperature) emerged as the most effective strategy, provided that a cryoprotectant was included. Sucrose‐stabilized freeze‐dried samples stored at −20°C retained transfection efficiency and resulted in even higher MFI than freshly prepared samples at Day 0, suggesting enhanced protein translation post‐reconstitution. Trehalose‐containing samples also maintained transfection efficiency, though their MFI was approximately 50% lower than that of sucrose‐containing samples counterparts, yet they were comparable to fresh controls.

### 2.7 Physicochemical and Structural Property Evaluations Expose Nanostructural and mRNA Retention Instabilities in Response to Low‐Temperature Storage

**FIGURE 7:**
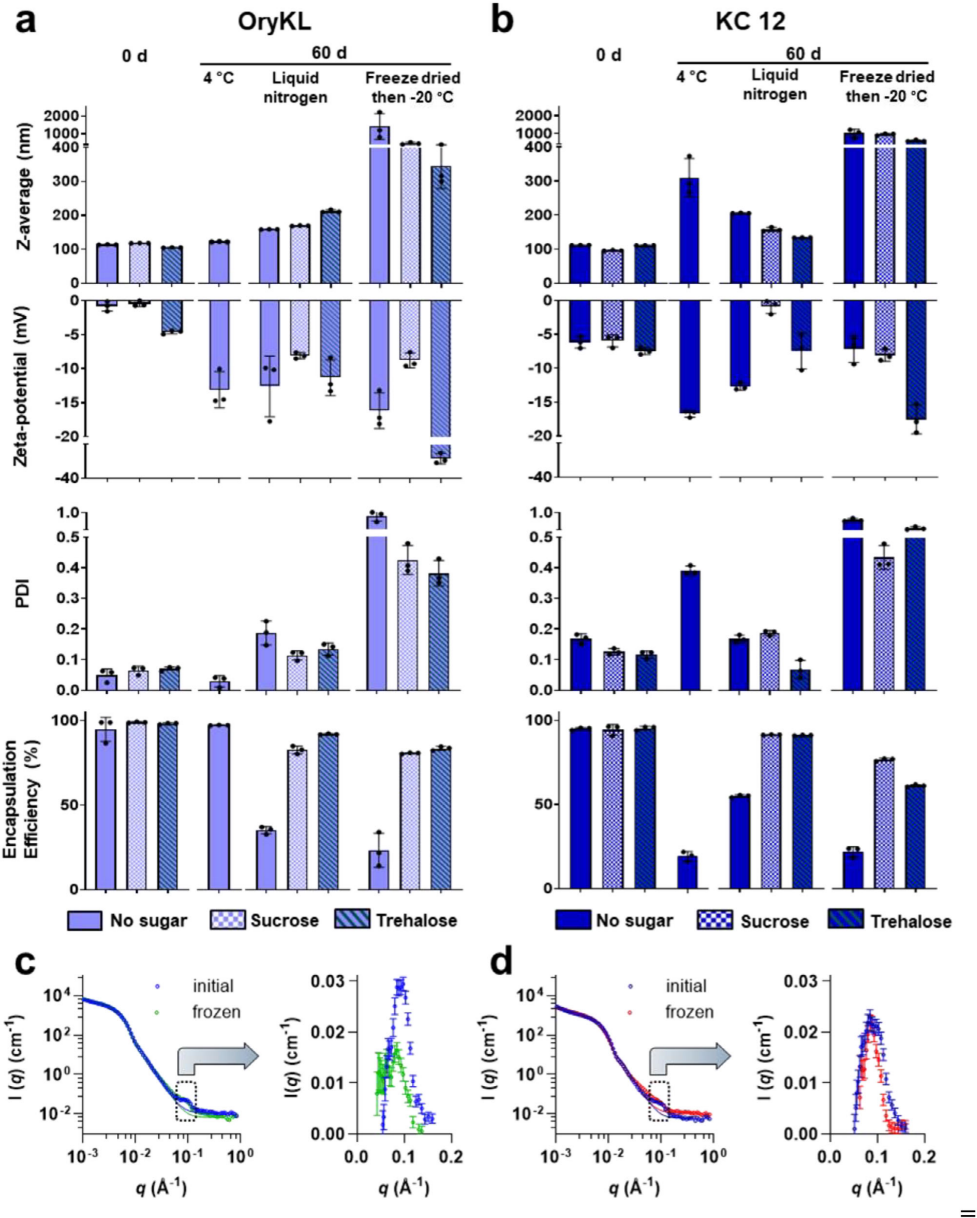
Effects of low‐temperature stress and storage on LNPs. Physicochemical properties and encapsulation efficacy of (a) OryKL and (b) KC 12 LNPs at 0 d and after 60 d storage at 4°C, liquid nitrogen, or freeze dried then stored at −20°C with or without cryoprotectant. Hydrodynamic diameter, polydispersity index (PDI), and zeta potential were measured using DLS and encapsulation efficiency (EE) was measured using RiboGreen assay. Bar graphs are shown as mean ± SD from independent experiment (*n* = 3). SANS data on (c) OryKL and (d) KC 12 LNPs before and after freezing for 24 h. Boxed region in black highlights the predominant feature at *q* ≈ 0.1 Å^−1^ that changed after freezing and the plots pointed by the arrows are the respective isolated SANS intensities within the feature region (0.5−1.5 Å^−1^), showing the shift in the peak position and change in overall scattering signals. Fitting parameters and calculated values for the full SANS patterns and isolated SANS intensities are available in Tables S1 and S2.

To better understand these performance differences, we characterized structural and functional integrity of stored OryKL using DLS (particle size and PDI), ELS (zeta potential), and encapsulation efficiency (mRNA retention) (Figure 7a). As a control to determine the effect of sterol substitution, we also performed parallel experiments on a KC2‐cholesterol LNP system (Figure 7b, referred to as KC 12, consistent with previous sections). Similarly, both OryKL and KC 12 stored in liquid nitrogen with or without cryoprotectants exhibited the least change in average particle size and PDI. All freeze‐dried samples appeared aggregating, indicating that DLS measurement may not reliably reflect structural integrity post‐lyophilization. Significant drop in EE values (from ∼90% to 30%–40%) was observed in cryoprotectant‐free samples stored in liquid nitrogen or lyophilized. In contrast, freeze‐dried samples with sucrose or trehalose retained approximately 80% of their mRNA payload, highlighting the critical role of cryoprotectants in maintaining LNP integrity during low‐temperature storage. One prominent disparity between the two sterol‐based systems lies in their structural integrity during storage at 4°C. KC 12 samples exhibited a marked increase in average particle size and PDI, along with a significant drop in EE% (∼20%), whereas the physiochemical properties and EE of OryKL were maintained after 60 days of storage at 4°C. Furthermore, total mRNA concentration remained stable for OryKL during storage at 4°C (Figure S15), confirming that the observed EE was not driven by degradation of unencapsulated mRNA. This indicates that the impact of sterol substitution may be more pronounced under prolonged storage at non‐cryogenic conditions in which LNPs remain in hydrated, partially dynamic state. The observed discrepancy between particle size and biological function (particularly in 4°C‐stored samples) suggests that factors beyond gross size changes contribute to LNP performance. Subtle alterations in internal structure or surface lipid arrangement may impact mRNA release and translation despite the preserved size and encapsulation efficiency, offering important directions for future mechanistic investigations.

### 2.8 Small‐Angle Neutron Scattering Unveils Internal Architectural Changes in OryKL Under Low‐Temperature Storage

To explore structural changes that may occur in OryKL during freezing that may impact their performance as mRNA delivery systems, we conducted small‐angle neutron scattering (SANS) experiments before and after a 24‐h freezing period. SANS is a powerful colloid characterization technique for probing the nano‐ and ultrastructure of materials (1–500 nm), and when combined with appropriate model fitting, can yield insights into particle size, shape, and structural arrangement [68]. In the context of LNPs, SANS has been successfully applied to characterize both overall geometry and subtle structural features, including internal lipid phases and mRNA localization [56, 69]

SANS patterns of eGFP mRNA‐loaded OryKL (Figure 7c) were fitted reasonably well using a core–shell model (CSM) both before and after freezing, indicating the preservation of a distinct internal core and external shell structure. This structural stability is consistent with cryoTEM observations and typical of mRNA‐loaded LNPs. No significant differences were observed, particularly in the low‐*q* region (< 0.1 Å^−1^) or the Guinier region, corresponding to the overall particle size. This similarity confirms that freezing does not significantly alter the overall size or morphology of OryKL, which is approximated to be 140 nm in diameter, based on CSM fitting (See parameters in Table S1), closely reflecting values obtained from DLS (Figure 2a) and cryoTEM (Figure 3c–e). However, changes were observed in the broad scattering feature in the high *q* range (0.5−1.5 Å^−1^, boxed region in Figure 7c), that is not captured by the core–shell model. This peak likely originates from smaller internal nanostructures within the LNPs and has been reported previously in mRNA‐loaded systems [56, 70, 71]. These features have been attributed to lipid self‐assembly in the form of a fluid isotropic phase or more ordered structure, potentially an irregularly shaped (branched) tubular or sheet‐like arrangement formed by the LNPs’ constituent lipids, water, and mRNA.

To quantify changes in this internal structure, scattering intensities within this *q* range were isolated and fitted after subtracting a power‐law background. The resulting intensity curves (Figure 7c, isolated high‐*q* intensities) reveal a peak broadening and peak shift from 0.092 to 0.077 Å^−1^ in OryKL, corresponding to an increase in the repeat distance from 6.8 nm to 8.1 nm. By comparison, KC 12 (Figure 7d, isolated high‐*q* intensities) exhibited a peak shift from 0.089 to 0.088 Å^−1^, corresponding to a modest increase in repeat distance from ∼7.0 to 7.2 nm, highlighting the more substantial structural rearrangement (below 10 nm) induced by γ‐oryzanol incorporation. Peak broadening and reduced intensity in this *q* range suggest a partial loss of these internal ordered domains, with remaining domains becoming more loosely packed or expanded, consistent with prior small‐angle X‐ray scattering studies on lyophilized mRNA LNPs [21]. In addition to the changes in the high‐*q* broad peak feature, a pronounced alteration in the power‐law exponent (Figure S16; Table S3) was observed in the intermediate *q* region (0.025–0.055 Å^−1^) of KC 12, decreasing from 3.58 to 2.70, compared with a more modest change in OryKL (from 3.64 to 3.25 after freezing). Together, these results indicate a length scale‐dependent response to freezing, whereby KC 12 preserves short‐range internal order but undergoes more extensive structural rearrangements at larger length scales. Such sterol‐dependent internal reorganization may influence the functional stability of stored mRNA LNPs.

More broadly, freezing‐induced changes in LNP internal organization, particularly alterations in short‐range ordering reflected by the high‐*q* broad peak, remain incompletely understood in the context of mRNA LNP stability and performance. In the present dataset, peak broadening is most consistent with reduced internal ordering (i.e., phase reorganization, lipid re‐packing, increased internal structural heterogeneity). Future studies integrating complementary structural approaches, including contrast variation‐SANS or high‐resolution synchrotron SAXS, will be valuable to further resolve these mechanisms. The present findings highlight the critical role of sterol chemistry in governing these structural responses, demonstrating that incorporation of non‐conventional sterols such as γ‐oryzanol induces distinct, length scale‐dependent internal rearrangements.

The present dataset provides a structural view of sterol‐dependent organization following preservation but was not designed to establish direct quantitative relationships with functional delivery outcomes. Clarifying how these nanoscale changes influence translation efficiency, stability, and in vivo performance will require studies that integrate controlled formulation variables with matched biological measurements across time. Developing this structure–function understanding represents an important direction for future investigation. Future mechanistic studies, including protein corona analysis, pharmacokinetics evaluations and cell type‐specific uptake profiling, will be important to better understand how sterol‐substitution influences in vivo behavior of LNP systems.

Although our data demonstrated that OryKL retained functional transfection after 60 days, defining true pharmaceutical shelf life will require longer observation periods, formal benchmarking against licensed formulations, and optimization of variables such as freeze–thaw handling and cryoprotectant strategies. In addition to functional readouts, direct structural assessment of mRNA integrity using electrophoretic or capillary‐based analyses will provide complementary confirmation of RNA stability during extended storage. Likewise, orthogonal particle characterization methods such as nanoparticle tracking analysis (NTA) or cryoTEM may offer additional insight into post‐reconstitution morphology and number‐based size distributions. Together, these studies will be essential for establishing robust translational design principles for sterol‐substituted LNP systems.

## 3 Conclusion

This study identified γ‐oryzanol and DLin‐KC2‐DMA based LNPs, OryKL, as a promising mRNA delivery platform with broad therapeutic potential. Through systematic combinatorial screening and multi‐stage evaluation, OryKL consistently demonstrated superior or comparable performance to conventional cholesterol‐based formulations. They exhibited excellent cytocompatibility and hemocompatibility across a wide dose range, high mRNA encapsulation efficiency, tunable particle size, and robust in vitro transfection capability.

In vivo, OryKL achieved efficient and cell‐specific mRNA delivery following intravenous administration, with preferential expression observed in the hepatocytes and tissue‐resident immune cells, while avoiding excessive uptake by Kupffer cells and limiting pro‐inflammatory cytokine responses. Immunogenicity and biosafety profiling revealed minimal immunostimulation, no histopathological abnormalities, and normal serum and hematological parameters, indicating a favorable safety profile that is well‐suited to chronic or immune‐sensitive therapeutic contexts.

Importantly, OryKL retained biological activity after long‐term storage, with freeze‐dried formulations stabilized by cryoprotectants, particularly sucrose, exhibiting preserved or even enhanced transfection efficiency and protein expression. Structural characterization via cryoTEM and SANS confirmed the stability of overall particle morphology but revealed freezing‐induced changes to internal nanostructures, offering mechanistic insights into how formulation and storage conditions may affect functional performance.

Collectively, these findings position OryKL as a versatile and translatable platform for advancing mRNA therapeutics beyond vaccines, especially for applications requiring safe, targeted, and storage‐stable delivery systems in chronic or systemic disease settings.

## 4 Experimental

### 4.1 Materials

All chemicals used in this study were used as received unless stated otherwise. Dulbecco's Modified Eagle Medium (DMEM), 0.05% trypsin−EDTA (TE, 1×) buffer, fetal bovine serum (FBS), *L*‐glutamine 200 mM (100×), and penicillin–streptomycin (P/S), 3‐(4,5‐dimethylthiazol‐2‐yl)‐2,5‐diphenyltetrazolium bromide (MTT), ribosomal RNA standard (100 µg mL^−1^ in TE buffer), single stranded RNA (ssRNA) ladder, Quant‐iT RiboGreen RNA Assay Kit, TRIzol, eBioscience 1X RBC Lysis Buffer, Anti‐Mouse CD11b eFluor450 (Cat. No. 48‐0112‐82), Anti‐Mouse CD14 APC (Cat. No. 17‐0141‐82), and Anti‐Mouse CD68 eFluor660 (Cat. No. 50‐0681‐82) were purchased from Thermo Fisher Scientific (Scoresby, VIC, Australia); BUV395 Rat Anti‐Mouse CD45 (Cat. No. 564279), PerCP‐Cy5.5 Rat Anti‐Mouse CD3 (Cat. No. 560527), BV786 Hamster Anti‐Mouse CD11c (Cat. No. 563735) were purchased from BD Biosciences. PE/Cyanine7 anti‐mouse CD19 antibody (Cat. No. 115520) was obtained from BioLegend; Liberase Research Grade (Roche) was obtained from Merck‐Australia; Nano‐Glo Fluorofurimazine In Vivo Substrate (Cat. No. N41100) was purchased from Promega; Cationic ionizable lipids ALC‐315, C12‐200, DLin‐MC3‐DMA, and DLin‐KC2‐DMA were purchased from Broadpharm (San Diego, CA, USA), while cationic ionizable lipid SM102 was purchased from ABP Bioscience; cholesterol, β‐sitosterol, 1,2‐distearoyl‐sn‐glycero‐3‐phosphoethanolamine‐*N*‐[methoxy (polyethylene glycol)‐2000] (PEG2000‐DSPE), and 1,2‐distearoyl‐*sn*‐glycero‐3‐phosphocholine (DSPC) were purchased from Avanti Polar Lipid, Inc.; β‐stigmasterol, dimethylsulfoxide (DMSO), Triton X‐100, chloroform and isopropanol were purchased from Sigma‐Aldrich; γ‐oryzanol was purchased from Wako Pure Chemical Industries, Ltd., Osaka, Japan; RNA Loading Dye was purchased from New England Biolabs; and nuclease‐free water was purchased from Integrated DNA Technologies (IDT). iTaq Universal SYBR Green One‐Step Kit was purchased from Bio‐Rad. mRNA constructs coding for the enhanced green fluorescent protein, eGFP (990 nt, Cap1 5’ capping, N1‐methyl‐pseudouridine‐modified, 120 nt polyA tail), mCherry (981 nt, Cap1 5’ capping, N1‐pseudouridine‐modified, 121 nt polyA tail), NanoLuc (873 nt, Cap1 5’ capping, N1‐pseudmethylouridine‐modified, 126 nt polyA tail), and Cre recombinase (1335 nt, Cap1 5’ capping, N1‐pseudomethyluridine‐modified, 126 nt polyA tail) were designed and synthesized by BASE mRNA Facility.

### 4.2 Pre‐Formulation Lipid Mixture Preparation for Screening and Testing

A simplified fabrication process (modified drying–hydration–sonication technique) was initially employed. Briefly, ethanolic stock lipid solutions were mixed at a lipid molar ratio of 52.0:8.0:38.5:1.5 (ionizable lipid: DSPC: sterol: PEG2000‐DSPE) in sterile 3 mL screw‐cap glass vials, followed by vacuum desiccator drying, forming a uniform lipid film. The dried lipid film was then hydrated with phosphate‐buffered saline (PBS) and sonicated using a Misonix S‐4000 Sonicator for 1 min (10 s on/off cycles at 50% amplitude) over ice. The resulting dispersions were then collected and stored at 4°C for further testing. Note that DLin‐KC2‐DMA and DLin‐MC3‐DMA share a common structural backbone (DLin) and terminal dimethylamine (DMA) headgroup, with the “KC2” and “MC3” denoting distinct linker and hydrophobic tail chemistries that influence their pKa, biodegradability, and lipid packing properties. For this reason, the names of our LNPs are as follows: ionizable lipids ALC‐315 (ALC), C12‐200 (C12), DLin‐MC3‐DMA (MC3), DLin‐KC2‐DMA (KC2), and SM102 (SM); their matching sterol cholesterol (CHOL), β‐stigmasterol (STIG), β‐sitosterol (SITO), and γ‐oryzanol (ORY).

### 4.3 Size and Surface Charge Characterizations

For dynamic light scattering (DLS, for particle size distribution and polydispersity index (PDI)) and electrophoretic light scattering (ELS, zeta‐potential or surface charge), all samples were prepared by 100‐fold dilution with either room‐temperature ultrapure water or PBS, followed by measurements at 25°C using a Zetasizer Nano ZS (Malvern Panalytical).

### 4.4 CryoTEM

Quantifoil R1.2/1.3 300 mesh ultraAu grids were glow‐discharged (Pelco easiGlow, 15 mA, 90 s) mRNA‐LNP dispersions (3.0 µL) were deposited onto the discharged grids, followed by an automatic vitrification sequence using a FEI Vitrobot Mark IV (FEI Systems): blot‐drying at 4°C with relative humidity at 100%, and then plunge‐freezing in liquid ethane (−174°C) cooled down by liquid nitrogen. Frozen grids were stored in liquid nitrogen until cryoTEM observation to maintain the vitrified state and prevent structural changes or degradation of the samples. Cryo‐grids were loaded into a 12 cryo‐grid autoloader and then imaged using an FEI Talos Arctica Cryo‐TEM (Thermo Scientific) operating at 200 kV with a 50 µm C2 aperture. Micrographs were acquired using a bottom‐mounted Falcon III direct electron detector in counting mode at 75 000× nominal magnification.

### 4.5 Hemocompatibility Testing

Fresh human blood (4 mL) was collected from healthy volunteers, following the ethical guidelines approved by the Baker Heart and Diabetes Institute, Melbourne, Australia (Alfred Human Ethics, Project No: 58/24). The blood incubated in Triton X‐100 was used as a positive control for 100% hemolysis, whereas the blood incubated in PBS served as a negative control group. Various concentrations (0, 10, 20, 45, 85, and 170 µg mL^−1^) were prepared in triplicates. All samples were incubated at 37°C in an orbital shaker (150 rpm) for 1 h. Afterwards, 20 µL of each blood sample was mixed with 120 µL of cell pack fluid for complete blood count (Sysmex) analysis to measure white blood cell (WBC), red blood cell (RBC), and platelets (PLT). The remaining samples were then centrifuged (2000 rpm, 10 min), and 10 µL of plasma was carefully transferred to 96‐well plate and diluted with 90 µL PBS. Hemolysis was quantified via UV‐Vis spectrophotometry (BMG Labtech Fluostar Omega Microplate Reader) at 550 nm, using (Equation 1) to calculate hemolysis percentage:

(1) $$\begin{array}{cc} & \mathrm{Hemolysis}\left(\%\right)\\ & =\frac{\left(\mathrm{Absorbance}\;\mathrm{of}\;\mathrm{test}\;\mathrm{sample}-\mathrm{Absorbance}\;\mathrm{of}\;\mathrm{PBS}\;\mathrm{control}\right)}{\;\left(\mathrm{Absorbance}\;\mathrm{of}\;\mathrm{positive}\;\mathrm{control}-\mathrm{Absorbance}\;\mathrm{of}\;\mathrm{PBS}\;\mathrm{control}\right)}\\ & \;\times 100\% \end{array}$$

### 4.6 Cell Culture

CHO, HEK293T, and SVEC4‐10 cells were maintained in DMEM supplemented with 10% FBS, 1% P/S, and 1% L‐glutamine at 37°C in a humidified atmosphere (5% CO_2_).

### 4.7 Cell Viability Tests

CHO cells and HEK293T cells were seeded in a 96‐well plate (10^5^ cells in 100 µL cell medium per well) and incubated for 24 h at 37°C. After 24 h, the medium was replaced with serum free DMEM containing LNPs made described as above (LNP preparation for screening and testing) at different concentrations (0, 10, 20, 45, 85, and 170 µg mL^−1^) with serum‐free DMEM medium. The 96 wells plate was incubated for 24 h at 37°C. 3‐(4,5‐dimethylthiazol‐2‐yl)‐2,5‐diphenyltetrazolium bromide (MTT) solution was prepared by dissolving the yellow tetrazolium salt in PBS, 10 µL MTT solution (5 mg mL^−1^ in PBS) was added to each well and further incubated for 4 h at 37°C. The medium was subsequently removed, leaving the insoluble formazan crystals in the wells. 100 µL DMSO was added into each well to dissolve the formazan crystals. Absorbance was read at 570 nm (BMG Labtech FLUOstar Omega Microplate reader). Cell viability was calculated by Equation (2):

(2) $$\mathrm{Cell}\;\mathrm{viability}\left(\%\right)=\frac{\mathrm{Absorbance}\;\mathrm{of}\;\mathrm{treated}\;\mathrm{cultures}}{\mathrm{Absorbance}\;\mathrm{of}\;\mathrm{untreated}\;\mathrm{control}}\times 100\%$$

### 4.8 mRNA Encapsulation in LNPs via Microfluidic Mixing

After selecting optimal pre‐formulation lipid mixes, mRNA was incorporated into LNPs using a NanoAssemblr microfluidic mixer (Precision Nanosystems). Stock ethanolic lipid solutions (DSPC, PEG2000‐DSPE, sterol, and ionizable lipids) were warmed to room temperature and sonicated for dispersion to ensure homogeneity prior to use. All lipids were combined in a 1.5 mL Eppendorf tube, following the molar ratio of 52:8:38.5:1.5 (ionizable lipid: helper lipid: sterol: PEG‐lipid). The required amount of mRNA (N/P ratio = 4) and acetate buffer (25 mM, pH 4) were mixed in a separate tube. The lipid and mRNA dispersions were loaded into syringes through 18 G blunt needles. The syringes were then loaded into the NanoAssemblr and mixed at a flow rate ratio of 3:1 (aqueous‐to‐ethanolic) with three distinct total flow rates: 8, 10, or 12 mL min^−1^. The resulting LNP dispersions (∼1–2 mL) were dialyzed overnight using dialysis cassettes (10 kDa MWCO) against 400 mL PBS. This process typically yields LNP Samples were characterized by DLS and ELS to confirm particle size and distribution (See Section 4.3) and either used immediately or stored at 4°C for short‐term storage.

### 4.9 Encapsulation Efficiency

The Quant‐iT Ribogreen RNA Assay (Thermo Fisher Scientific) was employed to assess the encapsulation efficiency. A standard RNA solution (2 µg mL^−1^in 1× TE buffer) was prepared for the calibration curve. For each LNP sample, two sets of triplicates were prepared: 2% LNPs with Triton X‐100 (2 µL LNPs + 1 µL Triton X‐100 + 97 µL 1× TE buffer, to release encapsulated mRNA) and 2% LNPs without Triton X‐100 (2 µL LNPs + 98 µL 1× TE buffer, to detect free mRNA). RiboGreen reagent was diluted 200‐fold in the 1× TE buffer. Each sample (100 µL) was added to the wells, followed by 100 µL pre‐diluted Ribogreen RNA reagent. Fluorescence was measured on a BMG Labtech FLUOstar Omega Microplate Reader (excitation ∼480 nm, emission ∼520 nm). Readings for the Triton X‐100 set were corrected for dilution (multiplied by 100/92), and sample RNA concentrations were derived by linear interpolation from the standard curve. Encapsulation efficiency was determined by (Equation 3):

(3) $$\begin{array}{cc} & \mathrm{EE}\left(\%\right)\\ & =\frac{\left(\mathrm{reads}\;\mathrm{of}\;\mathrm{samples}\;\mathrm{with}\;\mathrm{Triton}-\mathrm{reads}\;\mathrm{of}\;\mathrm{samples}\;\mathrm{without}\;\mathrm{Triton}\right)}{\left(\mathrm{reads}\;\mathrm{of}\;\mathrm{samples}\;\mathrm{with}\;\mathrm{Triton}\right)}\\ & \;\times 100\% \end{array}$$

### 4.10 Transfection Efficiency

CHO were seeded in a 6‐well plate at a density of 150 000 cells per well for a minimal time of 24 h and until it reaches ≥80% confluency, which corresponds to approximately 0.8–1.0 × 10^6^ cells. The plate was incubated for 24 h in the incubator (37°C, 5% CO_2_, 95% humidity). After washing with 1 mL PBS, 1 mL of serum‐free DMEM containing various amounts of mRNA‐LNPs (10, 20, 50, 100, 200, and 500 ng mRNA per 10^5^ cells) was added to each well. After 24 h, cells were imaged by fluorescence microscopy (Olympus IX‐81) using a 488 nm excitation laser for eGFP. Fluorescence images were processed using Fiji [72]. Brightness and contrast adjustments were applied using linear scaling and were kept identical across all images within each experiment with no further transformations or corrections applied. Cells were then detached with trypsin, pelleted, resuspended in 300 µL PBS, and analyzed for eGFP expression by flow cytometry (BD Fortessa). FlowJo software was used for data processing.

### 4.11 In Vitro Immune Response

SVEC4‐10 cells were cultured and transfected following the methods in Section 4.10. SVEC4‐10 cells were seeded in a 6‐well plate until ≥80% confluency. After washing with 1 mL PBS, 1 mL of serum‐free DMEM containing mRNA‐LNPs with 200 ng mRNA per 10^5^ cells was added to each well. The plate was incubated for 24 h in the incubator (37°C, 5% CO_2_, 95% humidity). Cells (1 × 10^6^ cells) were processed 24 h post‐transfection by lysing in 1 mL TRIzol Reagent. After 5 min incubation, 200 µL chloroform was added per 1 mL TRIzol, and samples were shaken vigorously, then centrifuged at 15,000×g for 15 min at 4°C. The upper aqueous phase was transferred to a new tube, mixed 1:1 with isopropanol, and centrifuged at 15,000×g for 10 min at 4°C. RNA pellets were washed three times with 75% ethanol, air‐dried, and dissolved in 200 µL of RNase‐free water. Purity and concentration of extracted RNA were measured via NanoDrop (optimal A260/A280 ratio ∼2). cDNA synthesis and qPCR were carried out using the Bio‐Rad One‐Step Kit in a 384‐well format (MicroAmp EnduraPlate Optical 384‐Well). Primers for pro‐inflammatory cytokines (IL‐6), adhesion molecules (VCAM‐1, ICAM‐1), and anti‐inflammatory cytokines (e.g., IL‐10) were selected to assess immune responses. The Applied Biosystems QuantStudio 7 platform was used with GAPDH as the internal reference gene. Relative expression levels were determined using the 2^−ΔΔCt method.

### 4.12 In Vivo Biodistribution Studies

#### 4.12.1 Animal Experiments

All animal experiments were approved by the Alfred Plus Alliance Animal Ethics Committee (No. E/8196/2025/B and E/10630/2024/B) and conducted in accordance with institutional guidelines at the Baker Heart and Diabetes Institute, Melbourne, Australia. C57BL/6 wild‐type mice (20–25 g, approximately equal numbers of males and females) were obtained from the Alfred Medical Research and Education Precinct Animal Services. B6.Cg‐Gt(ROSA)26Sortm9(CAG‐tdTomato)Hze/J (Ai9) mice (8–10 weeks old, approximately equal numbers of males and females) were purchased from Jackson Laboratory and bred at the Alfred Medical Research and Education Precinct Animal Facility (AMREP). Mice were randomly assigned to treatment or control groups in a blinded manner. All experimental procedures, including dosing and outcome assessments, were performed under blinded conditions.

#### 4.12.2 Preliminary Experiments with mCherry mRNA‐Loaded OryKL

Mixed‐sex C57BL/6 mice received either PBS control or mCherry mRNA loaded‐LNPs via IP and IV injection. After 24 h, all mice were euthanized via IP injection of ketamine/xylazine (300 and 50 mg kg^−1^, respectively), followed by liver excision. Liver samples were then processed for flow cytometry analysis (mCherry expression at 561 nm).

#### 4.12.3 Organ‐Level Biodistribution Studies using NanoLuc mRNA Reporter System

Mixed‐sex C57BL/6 mice received either OryKL encapsulating 10 µg NanoLuc mRNA or an equivalent volume of PBS via IV administration, corresponding to a dose of 0.5 mg kg^−1^. After 24 h, mice received 100 µL (0.46 µmol) IP injection of Nano‐Glo Fluorofurimazine In vivo substrate (Promega), freshly prepared in 1 mL sterile PBS. After 15 min, all mice were euthanized via IP injection of ketamine/xylazine. Major body organs (liver, spleen, lungs, kidneys, and heart) were collected for further analysis. The whole organ fluorescence was measured using an IVIS Lumina III imaging system and quantified using LivingImage Software.

#### 4.12.4 Organ‐ and Cell‐Level Biodistribution in Ai9 Mice

Mixed‐sex Ai9 mice were intravenously injected with Cre‐recombinase mRNA‐loaded LNPs at 0.5 mg kg^−1^ mRNA dose via tail vein injection, or PBS as a negative control. After 72 h, mice were humanely killed using IP injection of pentobarbitone (300 mg kg^−1^). Blood and major body organs (liver, spleen, lungs, kidneys, lymph nodes, and heart) were collected for further analysis. The whole organ fluorescence was measured using an IVIS Lumina III imaging system and quantified using LivingImage Software.

Blood samples were treated with red blood cells (RBCs) lysis buffer for 5 min on ice and then blood cells were washed in ice‐cold PBS via centrifugation at 400 g for 5 min. Single‐cell suspensions of lymph nodes and spleens were obtained using 70‐µm filters. Splenic cells were treated with RBCs lysis buffer for 5 min and then washed in ice‐cold PBS as mentioned before. Liver tissue was minced using a sterile scalpel blade and digested in a 100 µg mL^−1^ solution of Liberase at 37°C for 1 h. After neutralization with 10% FBS‐containing buffer, supernatant was removed using centrifugation at 400 g for 5 min. Cells were resuspended in RBCs lysis buffer for another 5 min at 4°C, then washed and passed through a 40‐µm filter to obtain single cell suspension.

Single cell suspensions were stained with a panel of anti‐mouse antibodies (see Section 4.1) at 1:300 dilution for the following markers: CD45, CD11b, CD11c, CD3, CD19, and CD14 or CD68. Data was acquired using a Cytek Aurora flow cytometer and analyzed using SpectroFlo Software. Cell populations were identified as: immune cells (CD45^+^), myeloid cells (CD45^+^ CD11b^+^), dendritic cells (CD45^+^ CD11b^+^ CD11c^+^), macrophages (CD45^+^ CD11b^+^ CD68^+^), monocytes (CD45^+^ CD11b^+^ CD14^+^), T cells (CD45^+^ CD11b^−^ CD3^+^), B cells (CD45^+^ CD11b^−^ CD19^+^), and liver cells as (CD45^−^ population in liver single cell suspensions). Gating strategy for the flow cytometry experiments is shown in Figure S17.

### 4.13 In Vivo Biosafety

#### 4.13.1 Immune Response by qPCR

Briefly, ∼25 mg liver tissue samples from Section 4.12.4 were lysed in 1 mL TRIzol Reagent. Subsequent RNA extraction, and qPCR analysis were performed following the same procedures described in Section 4.11. Gene expression of pro‐inflammatory cytokines (IL‐1β, IL‐6, TNF‐α, IFN‐γ), adhesion molecules (e.g., ICAM‐1), and anti‐inflammatory cytokines (e.g., IL‐10) was quantified using the Applied Biosystems QuantStudio 7 platform, with GAPDH as the internal reference gene. Relative expression levels were determined using the 2^−ΔΔCt method.

#### 4.13.2 Histological Analysis

Mouse tissues (from Section 4.12.4) were harvested and embedded in OCT (Tissue‐Tek, Sakura Finetek, USA), cryopreserved on dry ice, and stored at −80°C. The tissues were then cryosectioned 6 mm thickness onto superfrost glass slide using Epredia HM525NX Cryostat. For H&E staining, the tissue sections were equilibrated to room temperature for 30 min, followed by a 5 min incubation in PBS (Invitrogen, USA) to remove OCT. The sections were then fixed in formalin solution (Sigma‐Aldrich, USA) for 4 min, then washed in deionized water for 4 min. The tissues were stained with Harris Haematoxylin solution (Amber Scientific, Australian Biostain, South Australia) solution for 10 s and then rinsed in tap water until the water becomes colorless. The tissues were then incubated in Scott's tap water for 10 s and rinsed in running tap water for 5 min. For Eosin staining, tissues were incubated in Eosin 1% Alcoholic solution (Amber Scientific, Australian Biostain, South Australia) for 2 min. To differentiate the staining, the tissue sections were then dipped 10 times in 95% ethanol, followed by dehydration in 100% ethanol twice for 3 min, and two rinses in xylene (Sigma‐Aldrich, USA), each for 5 min. Finally, the slides were coverslipped using DPX Mountant (Sigma‐Aldrich, USA) and then imaged using an Olympus BX43 Microscope at 200× magnification.

### 4.14 Storage Stability Test

mRNA‐LNPs formulations (OryKL and KC 12 LNPs) were synthesized, diluted in PBS (No sugar, N), 20% w/v sucrose (SU), or 20% w/v trehalose (TR), aliquoted into 1.5 mL Eppendorf tubes or 2.0 mL cryo‐vials, and then subjected to three storage conditions: (1) 4°C (F) storage—only PBS diluted samples; (2) liquid nitrogen (LN) storage—all samples; and (3) −20°C for samples after lyophilization (FD). Prior to size measurements and transactions, LN‐stored samples were first rapidly thawed in a 25°C water bath, while lyophilized samples were reconstituted in deionized water. Transfection efficiency (Section 4.10) was assessed using CHO cells with the characteristics of mRNA‐LNPs measured at one and two months of storage under these conditions.

### 4.15 SANS Measurements

SANS experiments were performed using the Bilby SANS [73] beam line at Australian Centre for Neutron Scattering, Australian Nuclear Science and Technology Organisation (ANSTO), Lucas Heights, Australia. OryKL and KC 12 LNPs, prepared as described in Section 4.8, were dialyzed in 100 mM PBS (pH 7.4) made with deuterated water to reduce background signals and establish a strong contrast between the fully hydrogenous LNP components and the deuterated dispersing medium. For SANS measurements, the sample dispersions (final lipid concentratio*n* = 4 mg mL^−1^) were loaded into 300 µL Hellma cells with a 1 mm path length and mounted in a 16‐position sample changer. SANS measurements were conducted in time‐of‐flight mode with the incident neutron wavelength of 3–18 Å at ambient temperature (20°C). The raw scattering counts were collected on the main detector at a sample–detector distance of 18 m, combined with a pair of curtain detectors at 1.5 and 2.5 m. The data were reduced using the Mantid package [74], resulting in radially averaged intensity data *I*(*q*), where the scattering vector *q* is defined as:

(4) $$q=\frac{4\pi }{\lambda }\sin\frac{\theta }{2}$$

with λ the incident neutron wavelength and θ the scattering angle. Absolute intensity scaling was achieved based on an empty beam transmission measurement, and the simultaneous *q* range was 0.001–0.84 Å^−1^. For background subtraction, an empty cell measurement was used. The terms “intensity” or “scattering intensity” in this work refer to the macroscopic differential scattering cross‐section or volume‐specific differential scattering cross‐section (denoted as dΣ/dΩ), with the sample dispersion volume as the reference volume. All background‐subtracted SANS data were fitted using core–shell sphere, power‐law (for power‐law exponent changes in the intermediate *q* range), and Gaussian model (for the high‐*q* feature in each pattern) using SasView software (https://www.sasview.org).

### 4.16 Statistical Analysis

All experimental data were analyzed and plotted using GraphPad Prism (version 10.1.2). Grubb's test was used to detect and exclude outliers. For comparisons between two groups (e.g., one LNP formulation versus control), an unpaired two‐tailed *t*‐test was used. For experiments involving more than two groups, a one‐way analysis of variance (ANOVA) followed by Tukey's multiple comparisons test was conducted to determine specific group differences relative to negative control. For experiments involving two independent variables, such as LNP type and concentration, a two‐way ANOVA with Geisser‐Greenhouse correction was employed to evaluate the main effects and potential interaction between variables. Tukey's post hoc test was used for multiple comparisons. *p*‐value was set at 0.05 with significance levels denoted as: *p < 0.05; **p < 0.01; ***p < 0.001; ****p < 0.0001.

## Conflicts of Interest

Some authors are inventors listed on patent applications filed by their institution related to aspects of the technologies described in this manuscript. The authors declare no conflicts of interest.

## Supporting information




**Supporting File**: smll73095‐sup‐0001‐SuppMat.docx.

## Data Availability

The data that support the findings of this study are available in the supplementary material of this article.
