# Supplementary material for: Innovative γ‐Oryzanol and KC2 Based Lipid Nanoparticles: OryKL Platform Provides Safe and Efficient In Vivo mRNA Delivery
Source: Small. 2026 Mar 18;22(26):e11946. doi: 10.1002/smll.202511946 (PMC13155043; doi:10.1002/smll.202511946)
Supplement: Supplementary file 1 — Supporting File: smll73095‐sup‐0001‐SuppMat.docx. [file SMLL-22-e11946-s001.docx]

**SUPPORTING INFORMATION**


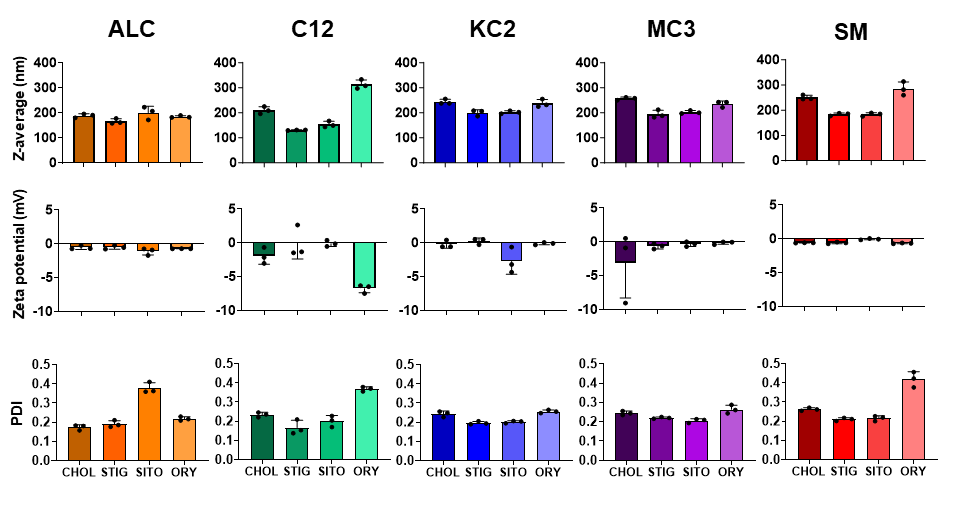


**Figure S1. *Physicochemical characterization of pre-formulation lipid mixtures used for MTT cytotoxicity and hemocompatibility screening.*** 20 pre-formulation lipid mixtures were formulated via a sonication-based method, and their particles were analyzed for hydrodynamic diameters (Z-average), zeta potential, and polydispersity index (PDI) via dynamic light scattering (DLS). Bar graphs are shown as mean ± SD from independent experiment (*n* = 3).


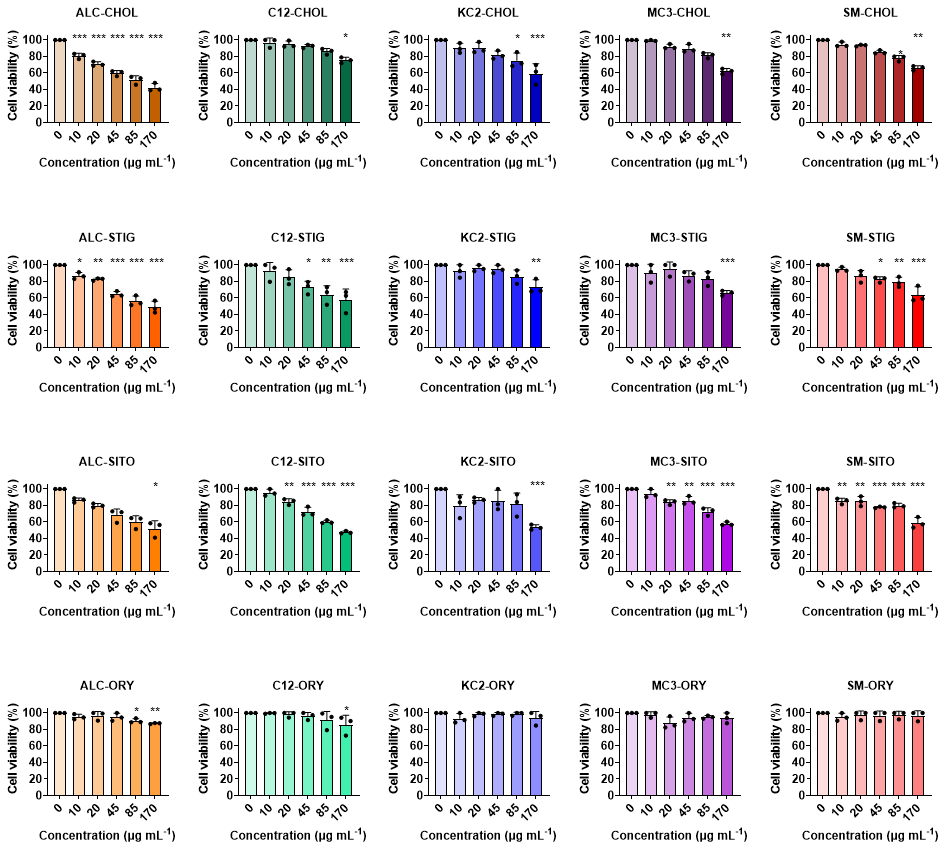


**Figure S2. *Cell viability of CHO cells following treatment with various pre-formulation lipid mixtures.*** MTT cell viability assessment using CHO cells following 24 h exposure to 20 different pre-formulation lipid mixtures across different concentrations ranges. Values are expressed as a percentage relative to the PBS control (0 µg mL^−1^, 100% viability). Bar graphs are shown as mean ± SD from independent experiment (*n* = 3). Statistical significance was tested using one-way ANOVA, followed by Dunnett's multiple comparisons test (comparing all treatment groups). Statistical comparisons reflect differences between lipid mixture treatment and control (PBS); **p* < 0.05, ***p* < 0.01, ****p* < 0.001.


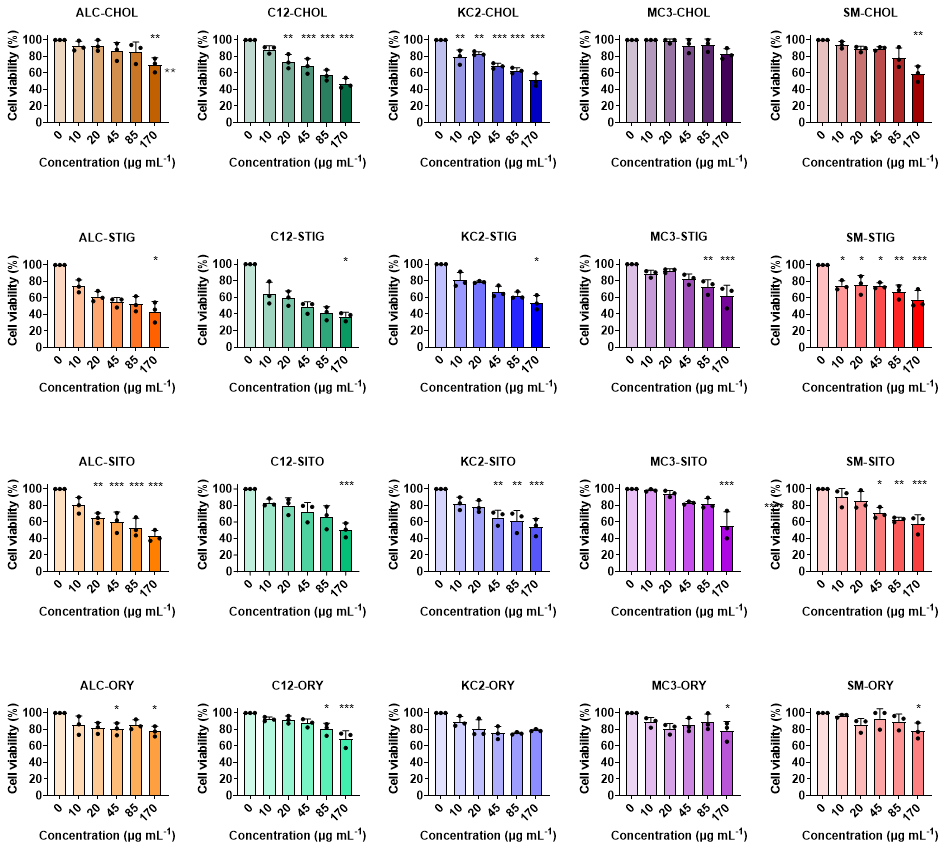


**Figure S3. *Cell viability of HEK293T cells following treatment with various pre-formulation lipid mixtures.*** MTT cell viability assessment using HEK293T cells following 24 h exposure to 20 different pre-formulation lipid mixtures across different concentration ranges. Values are expressed as a percentage relative to the PBS control (0 µg mL^−1^, 100% viability). Bar graphs are shown as mean ± SD from independent experiment (*n* = 3). Statistical significance was tested using one-way ANOVA, followed by Dunnett's multiple comparisons test (comparing all treatment groups). Statistical comparisons reflect differences between lipid mixture treatment and control (PBS); **p* < 0.05, ***p* < 0.01, ****p* < 0.001.


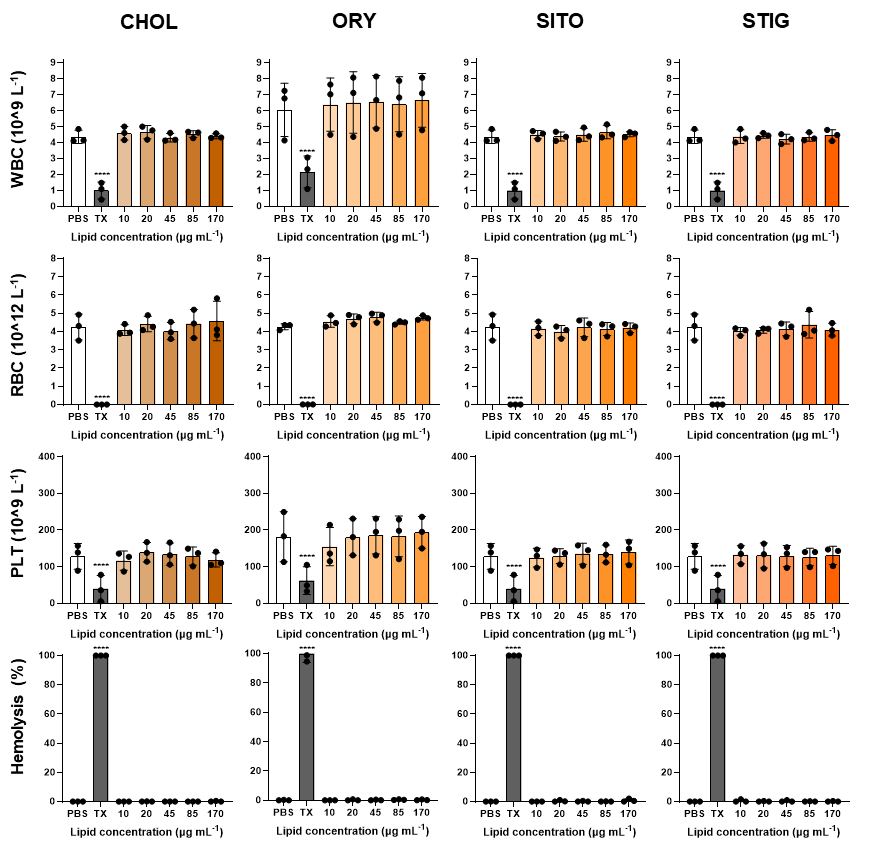


**Figure S4. *Hemocompatibility assessment of ALC-315-based pre-formulation lipid mixtures.*** Human whole blood was incubated with pre-formulation lipid mixtures for 1 h at 37°C, and hemolysis was quantified by measuring absorbance of released hemoglobin. Bar graphs are shown as mean ± SD from independent donors (*n* = 3). Triton X-100 was used as a positive control to indicate complete hemolysis. Statistical significance was tested using one-way ANOVA, followed by Dunnett's multiple comparisons test (comparing all treatment groups). Statistical comparisons reflect differences between lipid mixture treatment and control (PBS); *****p* < 0.0001.


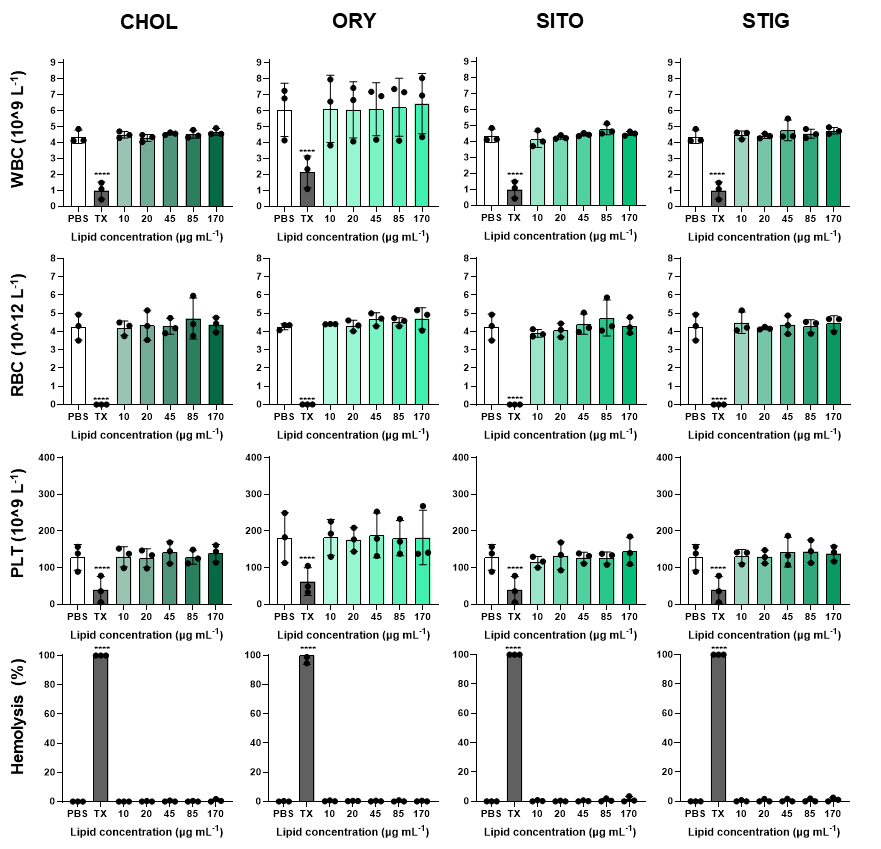


**Figure S5. *Hemocompatibility assessment of C12-200-based pre-formulation lipid mixtures.*** Human whole blood was incubated with pre-formulation lipid mixtures for 1 h at 37°C, and hemolysis was quantified by measuring absorbance of released hemoglobin. Bar graphs are shown as mean ± SD from independent donors (*n* = 3). Triton X-100 was used as a positive control to indicate complete hemolysis. Statistical significance was tested using one-way ANOVA, followed by Dunnett's multiple comparisons test (comparing all treatment groups). Statistical comparisons reflect differences between lipid mixture treatment and control (PBS); *****p* < 0.0001.


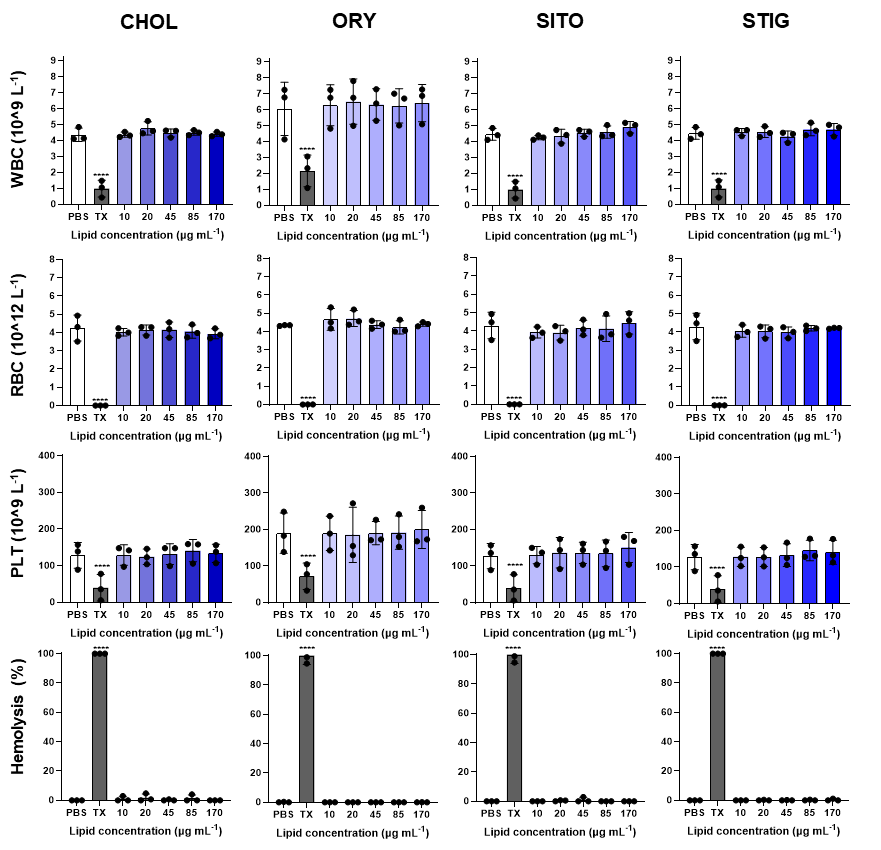


**Figure S6. *Hemocompatibility assessment of DLin-KC2-DMA-based pre-formulation lipid mixtures.*** Human whole blood was incubated with pre-formulation lipid mixtures for 1 h at 37°C, and hemolysis was quantified by measuring absorbance of released hemoglobin. Bar graphs are shown as mean ± SD from independent donors (*n* = 3). Triton X-100 was used as a positive control to indicate complete hemolysis. Statistical significance was tested using one-way ANOVA, followed by Dunnett's multiple comparisons test (comparing all treatment groups). Statistical comparisons reflect differences between lipid mixture treatment and control (PBS); *****p* < 0.0001.


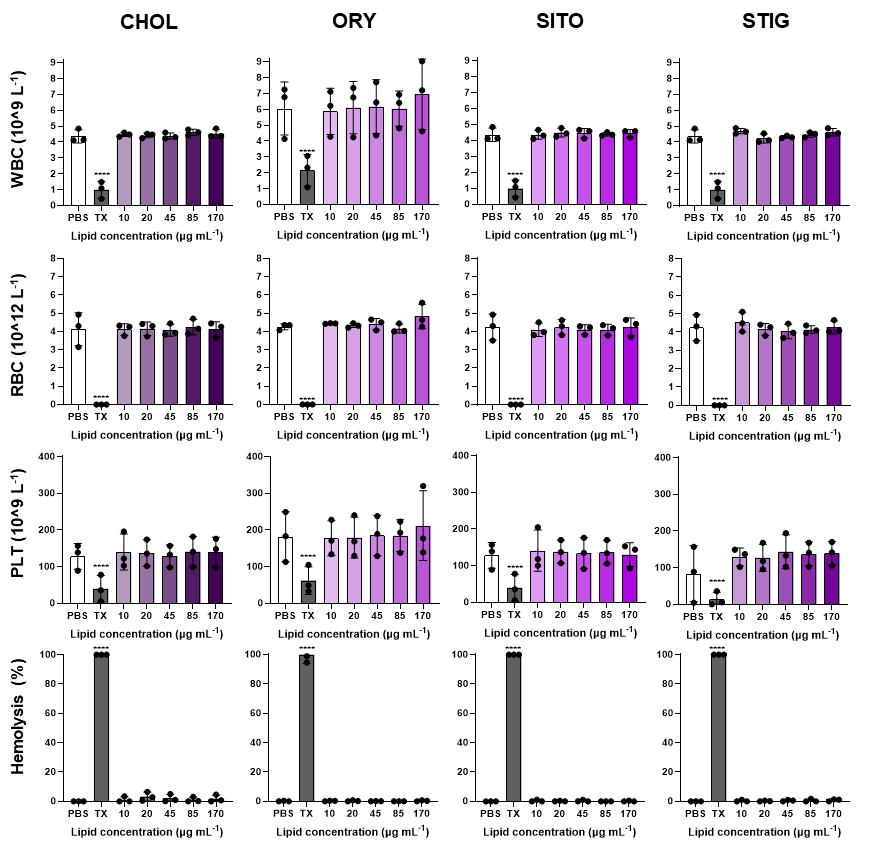


**Figure S7. *Hemocompatibility assessment of DLin-MC3-DMA-based pre-formulation lipid mixtures.*** Human whole blood was incubated with pre-formulation lipid mixtures for 1 h at 37°C, and hemolysis was quantified by measuring absorbance of released hemoglobin. Bar graphs are shown as mean ± SD from independent donors (*n* = 3). Triton X-100 was used as a positive control to indicate complete hemolysis. Statistical significance was tested using one-way ANOVA, followed by Dunnett's multiple comparisons test (comparing all treatment groups). Statistical comparisons reflect differences between lipid mixture treatment and control (PBS); *****p* < 0.0001.


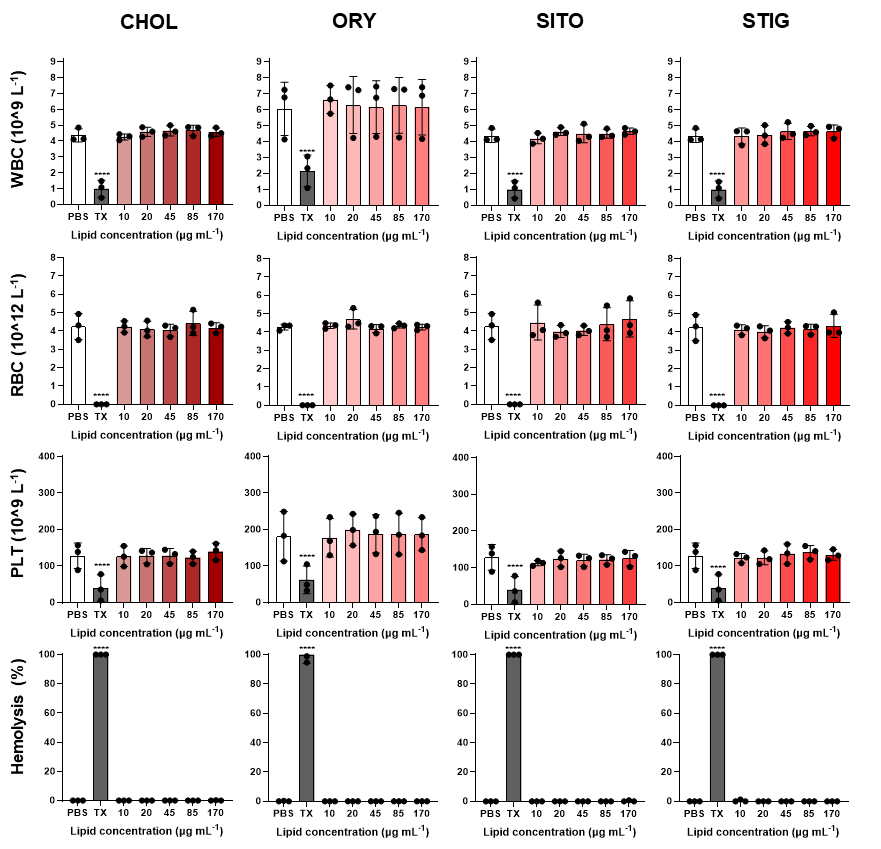


**Figure S8. *Hemocompatibility assessment of SM102-based pre-formulation lipid mixtures.*** Human whole blood was incubated with pre-formulation lipid mixtures for 1 h at 37 °C, and hemolysis was quantified by measuring absorbance of released hemoglobin. Bar graphs are shown as mean ± SD from independent donors (*n* = 3). Triton X-100 was used as a positive control to indicate complete hemolysis. Statistical significance was tested using one-way ANOVA, followed by Dunnett's multiple comparisons test (comparing all treatment groups). Statistical comparisons reflect differences between lipid mixture treatment and control (PBS); *****p* < 0.0001.


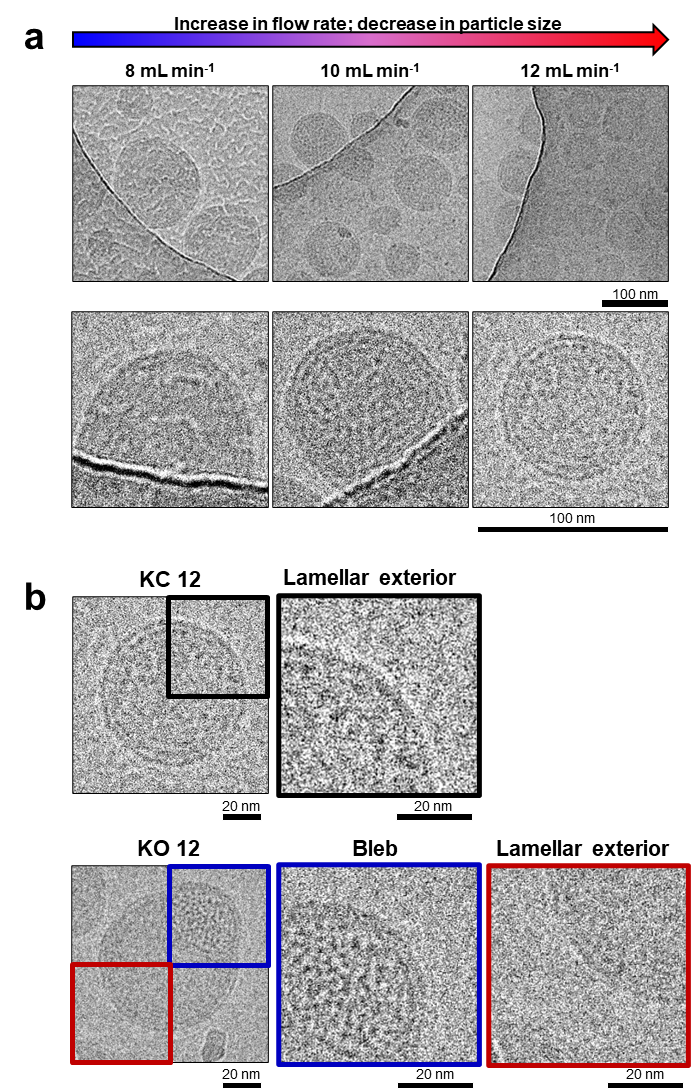


**Figure S9. *Impact of microfluidic mixing flow rate and sterols on the nanostructure of DLin-KC2-DMA based mRNA LNPs.*** Representative cryoTEM images depicting (**a**) the overall morphologies and internal structures of DLin-KC2-DMA-based mRNA LNPs prepared at different flow rates (8, 10, and 12 mL min^−1^). (**b**) CryoTEM images showing distinct structural features of KC 12 and KO 12 LNPs: lamellar exterior layers in both LNPs (black and red boxes) and blebs in KO 12 LNPs (blue box).


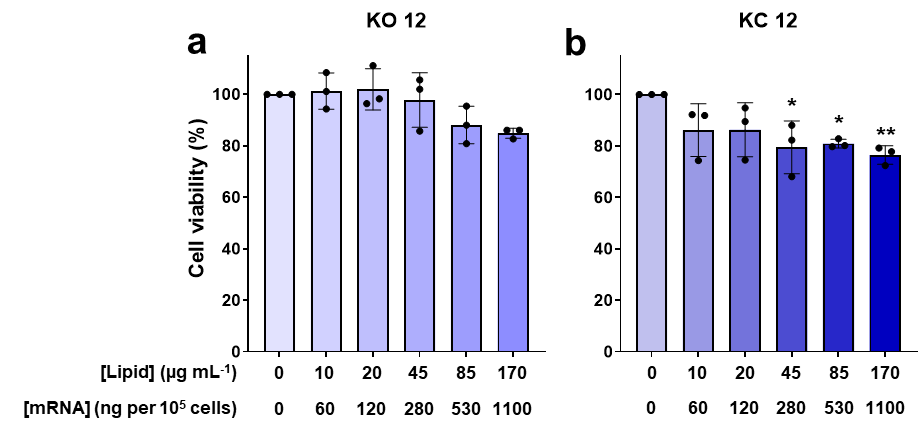


**Figure S10. *Cell viability of CHO cells following treatment with LNPs prepared via microfluidic mixing.*** MTT cell viability assessment using CHO cells following 24 h exposure to the two leading LNP formulations across different concentrations ranges: (**a**) KO 12 and (**b**) KC 12 LNPs. Values are expressed as a percentage relative to the PBS control (0 µg mL^-1^, 100% viability). Bar graphs are shown as mean ± SD from independent experiments (*n* = 3). Statistical significance was tested using one-way ANOVA, followed by Dunnett’s multiple comparisons test (comparing all treatment groups). Statistical comparisons reflect differences between LNP treated and control (PBS); **p* < 0.05, ***p* < 0.01.


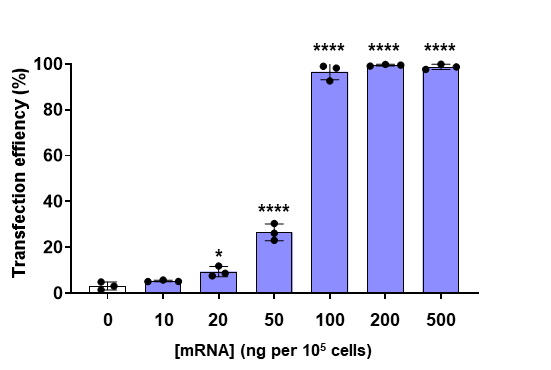


**Figure S11.** ***Transfection efficiencies of KO 12 at an extended dose range.*** CHO cells were treated with mRNA LNPs for 24 h at 10, 20, 50, 100, 200 and 500 ng per 10^5^ cells. Flow cytometry analysis showing the CHO cell population expressing eGFP, indicating transfection efficiency. Bar graphs are shown as mean ± SD from independent experiment (*n* = 3). Statistical significance was tested using one-way ANOVA, followed by Dunnett’s multiple comparison test (comparing all treatment groups). Statistical comparisons reflect difference between OryKL treated with control (PBS, 0 ng per 10^5^ cells). **p* < 0.05, *****p* < 0.0001.


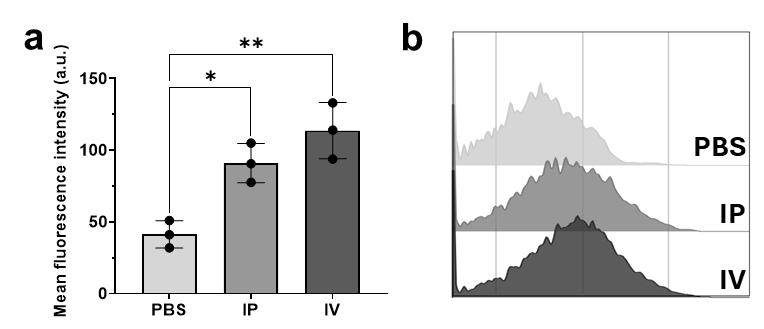


**Figure S12. *Preliminary in vivo evaluation of mCherry mRNA-loaded OryKL in mouse liver following different administration routes****.* Flow cytometry analysis was performed on isolated liver cells 24 h post-injection with OryKL encapsulating mCherry mRNA or PBS (control). (**a**) Mean fluorescence intensity data of mCherry in liver cell population, Bar graphs are shown as mean ± SD from independent mice (*n* = 3). Statistical significance was tested using one-way ANOVA, followed by Dunnett's multiple comparisons test (comparing all treatment groups). Statistical comparisons reflect differences between LNP treated and control (PBS); **p* < 0.05, ***p* < 0.01. (**b**) Overlaid histograms showing mean fluorescence intensity of mCherry in transfected liver cells following intravenous or intraperitoneal administration.


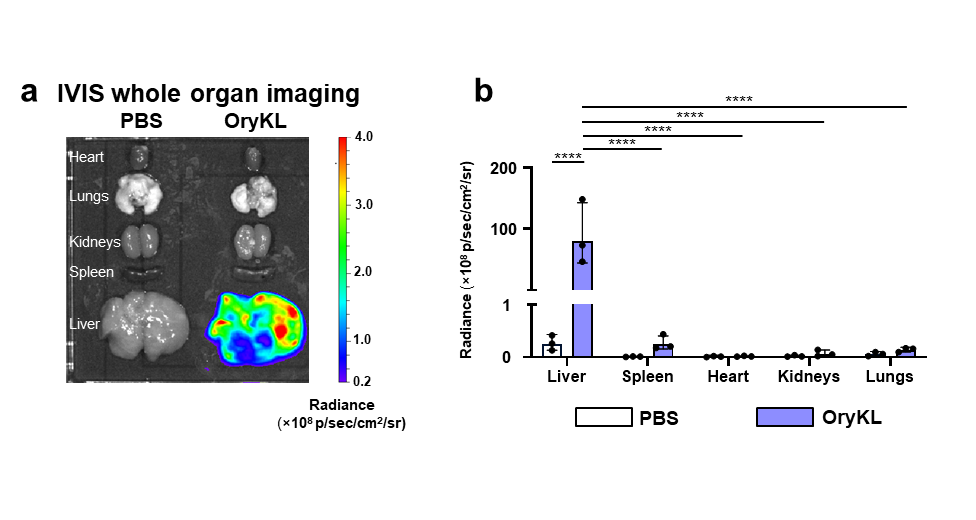


**Figure S13. *In vivo Nanoluc mRNA delivery and biodistribution of OryKL*.** (**a**) Representative IVIS bioluminescence photomicrographs of isolated major organs of C57BL/6 mice 24 h post-treatment with Nanoluc mRNA-loaded OryKL and with PBS (control). Color scale shows the bioluminescence signal intensities as radiance (in ×10^8^ p/s/cm²/sr). (**b**) Bar graphs showing the quantitative analysis using radiance in the bioluminescence photomicrographs of isolated major organs. Bar graphs are shown as mean ± SD from independent animals (*n* = 3), using two-way ANOVA (comparing means for all organs and treatment groups in **b**); *****p* < 0.0001.


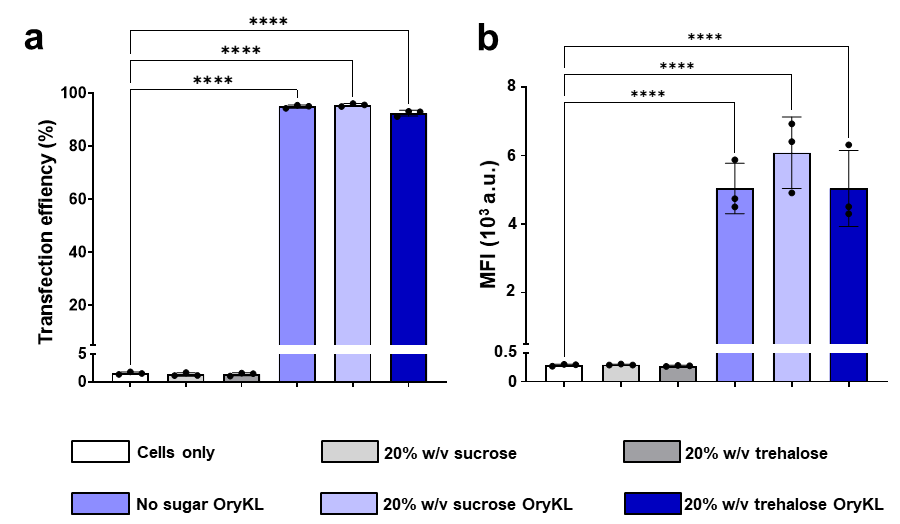


**Figure S14. *Evaluation of the impact of cryoprotectant addition on the transfection performance of eGFP mRNA loaded OryKL in CHO cells***. (**a**) Transfection efficiency and (**b**) MFI data on CHO cells 24 h post transfection with LNPs formulated with or without 20% (w/v) sucrose or trehalose. Bar graphs are shown as mean ± SD from independent experiment (*n* = 3). Statistical significance was tested using one-way ANOVA, followed by Dunnett's multiple comparisons test (comparing all treatment groups). Statistical comparisons reflect differences between LNP treatment and control (PBS); *****p* < 0.0001.


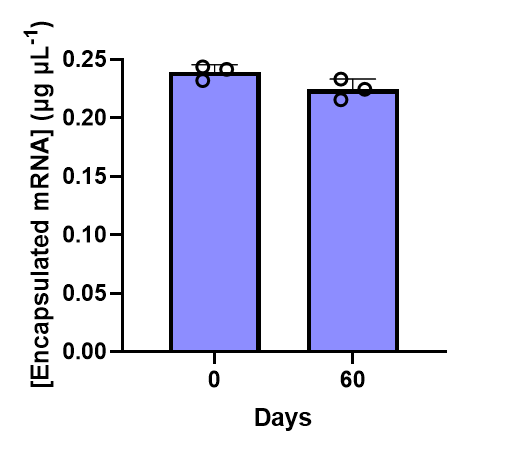


**Figure S15. *Stability of encapsulated mRNA content in OryKL during storage at 4°C*.** Encapsulated mRNA concentration was quantified using the RiboGreen assay. Bar graphs represent mean ± SD from independent experiments (*n* = 3). Statistical significance was assessed using an unpaired t-test comparing 0 d and 60 d; no significant difference was observed.

**
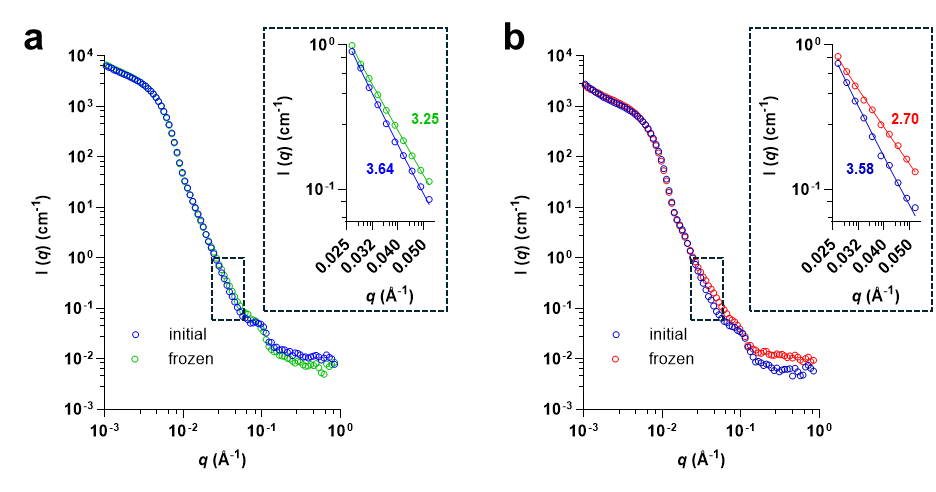
**

**Figure S16**. ***Power-law fitting of SANS data***. Local fitting of SANS data on (**a**) OryKL and (**b**) KC 12 LNPs in PBS (with 100% D_2_O) in the intermediate *q* region = 0.025–0.055 Å^−1^, highlighted by the boxed region in the full SANS patterns. Insets show the power law fitting (color-matched solid lines) with their respective fitting derived power-law exponents (See SasView parameters in Table S3).


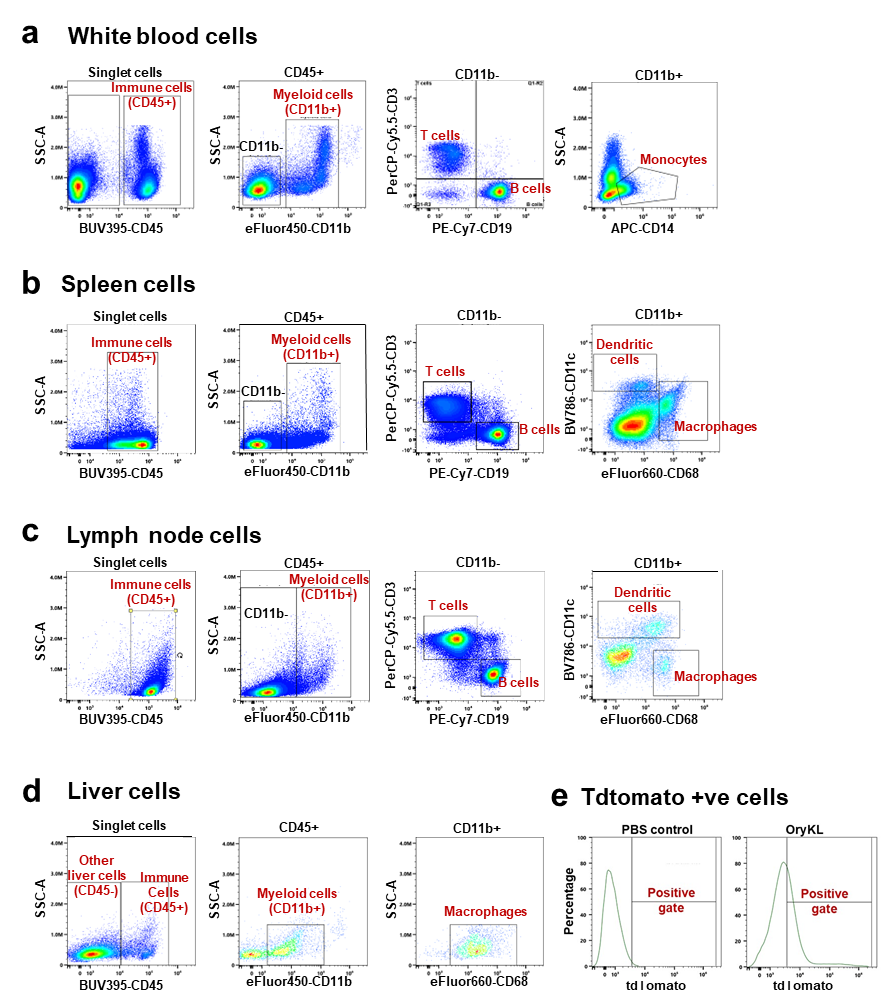


**Figure S17**. ***Gating strategy for respective cell populations in mouse***: (**a**) blood, (**b**) spleen, (**c**) lymph nodes, and (**d**) liver. Cells were first gated based on forward and side scatter (FSC-A vs SSC-A), followed by singlet selection identified using FSC-A vs FSC-H. (**e**) Representative histograms showing gating for tdTomato-positive cells.

**Table S1**. Fitting parameters for the core–shell sphere model fitting of SANS data on OryKL in PBS (with 100% D_2_O). Model fits are shown in Figure 7c and 7d in the Main Document.

| **Fitting parameter*** | **Initial state** | **After freezing** | **Initial state** | **After freezing** |
| --- | --- | --- | --- | --- |
| Background** | 0.008 (fixed) | 0.007 (fixed) | 0.006 (fixed) | 0.010 (fixed) |
| Scale** | 0.01 (fixed) | | | |
| Core radius (nm) | 43.57 (0.01) | 44.12 (0.01) | 31.48 (0.07) | 32.46 (0.07) |
| Thickness (nm) | 26.28 (0.01) | 26.89 (0.01) | 24.30 (0.10) | 25.45 (0.11) |
| Core SLD**  (×10^−6^ Å^−2^) | 0.12 (fixed) | | | |
| Shell SLD**  (×10^−6^ Å^−2^) | 6.22 (fixed) | | | |
| Solvent SLD** (×10^−6^ Å^−2^) | 6.35 (fixed) | | | |
| Radius polydispersity | 0.1 (fixed) | | | |
| Thickness polydispersity | 0.1 (fixed) | | | |
| χ^2^ | 1669 | 1842 | 837 | 1104 |

*****Fixed = fixed parameters; Numbers in parentheses indicate the SasView error values.

******Bases of values used: Background – model fitted value; scale – calculated total volume of all components used, incorporating total solvent volume changes during processing; core SLD value – based on preliminary data with core mainly composed of KC2 and ORY; shell SLD – based on preliminary data with shell mainly composed water (exchanged with surrounding dispersing medium).

Note: The model fitting was performed using single-contrast measurements. While this provides valuable structural insights, estimates may be further refined with complementary multi-contrast data.

**Table S2**. Fitting parameters for the Gaussian model fitting of background (power-law)-subtracted SANS data on OryKL in PBS (with 100% D_2_O).

| **Fitting parameter*** | **OryKL** | | **KC 12** | |
| --- | --- | --- | --- | --- |
|  | **Initial** | **After freezing** | **Initial** | **After freezing** |
| Background | 0.000 (fixed) | 0.000 (fixed) | 0.000 (fixed) | 0.000 (fixed) |
| Scale | 0.030 (fixed) | 0.016 (fixed) | 0.023 (fixed) | 0.022 (fixed) |
| Peak position (Å^−1^) | 0.092 (0.001) | 0.077 (0.001) | 0.089 (0.001) | 0.088 (0.001) |
| Correlation distance of peak position** (nm) | 6.8 | 8.1 | 7.0 | 7.2 |
| Sigma | 0.020 (0.001) | 0.024 (0.001) | 0.025 (0.001) | 0.017 (0.001) |
| χ^2^ | 1.7 | 1.3 | 0.5 | 0.9 |

*****Fixed = fixed parameters; Numbers in parentheses indicate the SasView error values.

******Calculated value from the model fitting-derived parameter.

**Table S3**. Fitting parameters for the power-law fitting of SANS data (intermediate *q* region = 0.025–0.055 Å^−1^) on OryKL and KC 12 LNPs in PBS (with 100% D_2_O).

| **Fitting parameter*** | **OryKL** | | **KC 12** | |
| --- | --- | --- | --- | --- |
|  | **Initial** | **After freezing** | **Initial** | **After freezing** |
| Background | 0.008 (fixed) | 0.007 (fixed) | 0.006 (fixed) | 0.010 (fixed) |
| Scale | 1.5 × 10^−6^  (1.6 × 10^−7^) | 1.5 × 10^−6^  (1.9 × 10^−7^) | 6.8 × 10^−6^  (6.1 × 10^−7^) | 4.2 × 10^−5^  (3.6 × 10^−6^) |
| Power law | 3.64 (0.03) | 3.25 (0.02) | 3.58 (0.04) | 2.70 (0.03) |
| χ^2^ | 2.5 | 1.5 | 4.0 | 2.6 |

*****Fixed = fixed parameters; Numbers in parentheses indicate the SasView error values.
